# In vivo adenine base editing of *PCSK9* in macaques reduces LDL cholesterol levels

**DOI:** 10.1038/s41587-021-00933-4

**Published:** 2021-05-19

**Authors:** Tanja Rothgangl, Melissa K. Dennis, Paulo J. C. Lin, Rurika Oka, Dominik Witzigmann, Lukas Villiger, Weihong Qi, Martina Hruzova, Lucas Kissling, Daniela Lenggenhager, Costanza Borrelli, Sabina Egli, Nina Frey, Noëlle Bakker, John A. Walker, Anastasia P. Kadina, Denis V. Victorov, Martin Pacesa, Susanne Kreutzer, Zacharias Kontarakis, Andreas Moor, Martin Jinek, Drew Weissman, Markus Stoffel, Ruben van Boxtel, Kevin Holden, Norbert Pardi, Beat Thöny, Johannes Häberle, Ying K. Tam, Sean C. Semple, Gerald Schwank

**Affiliations:** 1 University of Zurich, Institute for Pharmacology and Toxicology, Zurich, Switzerland; 2 Acuitas Therapeutics Inc., Vancouver, BC Canada; 3 Oncode Institute, Princess Máxima Center for Pediatric Oncology, Utrecht, Netherlands; 4 Functional Genomics Center Zurich, ETH Zurich/University of Zurich, Zurich, Switzerland; 5 Department of Biology, Institute for Molecular Health Sciences, ETH Zurich, Zurich, Switzerland; 6 Department of Pathology and Molecular Pathology, University Hospital and University of Zurich, Zurich, Switzerland; 7 Department of Biosystems Science and Engineering, ETH Zurich, Zurich, Switzerland; 8 Synthego Corporation, Redwood City, CA USA; 9 Department of Biochemistry, University of Zurich, Zurich, Switzerland; 10 Genome Engineering and Measurement Laboratory, ETH Zurich, Zurich, Switzerland; 11 Department of Medicine, University of Pennsylvania, Philadelphia, PA USA; 12 Division of Metabolism and Children’s Research Centre, University Children’s Hospital Zurich, Zurich, Switzerland; 13 Zurich Center for Integrative Human Physiology, Zurich, Switzerland; 14 Neuroscience Center Zurich, Zurich, Switzerland; 15 Institute of Molecular Life Sciences, University of Zurich, Zurich, Switzerland

**Keywords:** Targeted gene repair, Genetic engineering

## Abstract

Most known pathogenic point mutations in humans are C•G to T•A substitutions, which can be directly repaired by adenine base editors (ABEs). In this study, we investigated the efficacy and safety of ABEs in the livers of mice and cynomolgus macaques for the reduction of blood low-density lipoprotein (LDL) levels. Lipid nanoparticle–based delivery of mRNA encoding an ABE and a single-guide RNA targeting *PCSK9*, a negative regulator of LDL, induced up to 67% editing (on average, 61%) in mice and up to 34% editing (on average, 26%) in macaques. Plasma PCSK9 and LDL levels were stably reduced by 95% and 58% in mice and by 32% and 14% in macaques, respectively. ABE mRNA was cleared rapidly, and no off-target mutations in genomic DNA were found. Re-dosing in macaques did not increase editing, possibly owing to the detected humoral immune response to ABE upon treatment. These findings support further investigation of ABEs to treat patients with monogenic liver diseases.

## Abstract (web summary)

Base editors are effective and safe for cholesterol reduction in non-human primates.

## Main

Programmable CRISPR–Cas nucleases enable genome editing by generating double-stranded DNA breaks at the target locus^1^. For introducing precise changes at single-nucleotide resolution, induced DNA breaks require repair by exogenous donor templates via homologous recombination^1–4^. This process, however, is highly inefficient in post-mitotic cells^5,6^, and the use of targeted nucleases for gene editing therapies is, thus, limited to proliferating cells. Base editors are more recently developed genome engineering tools, where either a cytidine or an adenine deaminase is covalently linked to catalytically impaired Cas9. They convert C•T into T•A or A•T into G•C base pairs without the requirement of homology-directed repair and, therefore, enable precise and efficient editing in tissues with slow turnover rates, such as the liver^7–10^. Considering that most pathogenic point mutations are C•G to T•A conversions, ABEs are of particular interest for in vivo genome editing therapies^8^.

For clinical application of base editing, the potential generation of off-target mutations represents a major concern. Off-target mutations could be single guide RNA (sgRNA) dependent^11,12^ or sgRNA independent^13–15^ and are influenced by the levels and duration of base editor expression^16–18^. Thus, the risks of generating off-target mutations in therapies are likely to depend on the delivery method and dose. In addition, in vivo base editing has not yet been demonstrated in large animal models, and it remains unclear whether currently available delivery vectors are efficient enough to enable base editing in patients with therapeutic effects. In this study, we investigated the safety and efficacy of in vivo adenine base editing in the liver of mice and non-human primates (NHPs). We targeted *PCSK9* (proprotein convertase subtilisin/kexin type 9), which is primarily expressed in the liver and acts as a negative regulator of the LDL receptor^19^. Disrupting its function reduces blood LDL levels and represents a promising therapeutic approach for familial hypercholesterolemia with heterozygous loss-of-function mutations in *LDLR* or gain-of-function mutations in *PCSK9* (refs. ^20–22^). We report that lipid nanoparticle (LNP)-mediated delivery of ABE-encoding nucleoside-modified mRNA, together with a chemically stabilized sgRNA, enables efficient editing in mice and NHPs without inducing off-target mutations on genomic DNA.

## Results

### Inactivating PCSK9 by adenine base editing in mouse liver reduces blood LDL

**Fig. 1:**
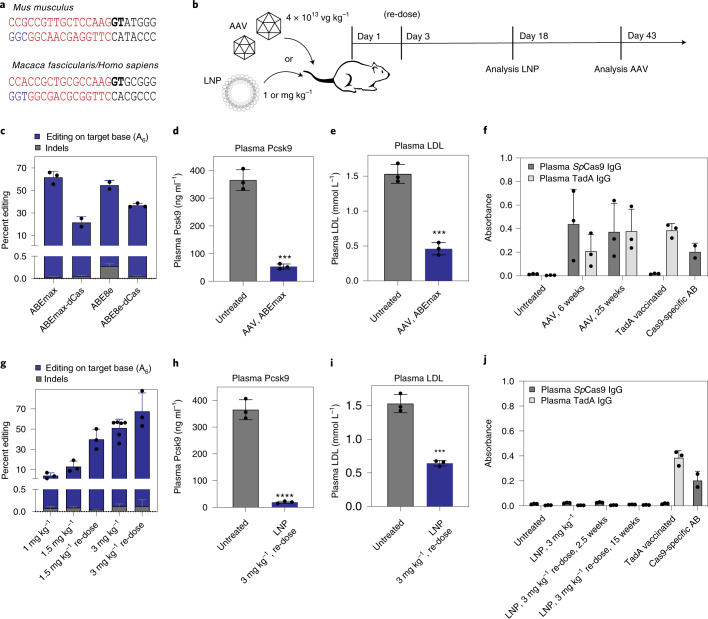
In vivo adenine base editing of the *Pcsk9* locus in the mouse liver. **a**, sgRNA target sequences in the mouse and macaque/human *PCSK9* gene. The exonic region (exon 1) is highlighted in red; the intronic region is highlighted in black. The GT splice donor recognition site is highlighted in bold letters. The NGG-PAM site is indicated in blue. **b**, Schematic outline of the mouse experiments. Vectors were administered intravenously via tail vein injection in adult C57BL/6J mice. **c**, Percent editing after treatment with different ABE variants. Insertions and deletions are summarized as ‘Indels’. Values represent mean ± s.d. of *n* = 3, *n* = 2, *n* = 2 and *n* = 3 animals. **d**, Plasma Pcsk9 protein levels as determined by ELISA. ***P = 0.0002. **e**, Plasma LDL cholesterol levels. ***P = 0.0003. **f**, Background-subtracted absorbance (*A*_450_ – *A*_540_) representing the relative amount of anti-Cas9- or anti-TadA-specific plasma IgG antibodies as determined by ELISA. Positive control: plasma of TadA vaccinated animals or commercial Cas9-specific antibody at a concentration of 200 ng ml^−1^ (*n* = 2). **g**, Percent editing after treatment with different doses of LNP-encapsulated mRNA/sgRNA. Insertions or deletions are summarized as ‘Indels’. **h**, Plasma Pcsk9 protein levels as determined by ELISA. ****P < 0.0001. **i**, Plasma LDL cholesterol levels. ***P = 0.0004. **j**, Background-subtracted absorbance (*A*_450_ – *A*_540_) representing the relative amount of anti-Cas9- or anti-TadA-specific plasma antibodies as determined by ELISA. Positive control: plasma of TadA vaccinated animals or commercial Cas9-specific antibody at a concentration of 200 ng ml^−1^ (*n* = 2). Unless otherwise stated, values represent mean ± s.d. of *n* = 3 animals. Means were compared using two-tailed unpaired *t*-tests. vg, vector genomes.

ABEs can be used to eliminate gene function by disrupting canonical splice sites^23,24^. To assess if this approach enables degradation of Pcsk9 mRNA and protein, we targeted several canonical *Pcsk9* splice sites in murine Hepa1-6 cells using adenine base editing (Supplementary Fig. 1a). The highest editing rates were observed at the GT splice donor site of murine *Pcsk9* intron 1 using sgRNA_mP01 (84 ± 4.6%; Supplementary Figs. 1b,c and 2a–c), leading to a substantial reduction in Pcsk9 mRNA and protein levels (Supplementary Fig. 2d,e). For the human *Pcsk9* splice donor site of intron 1, we observed similar editing efficiencies with the corresponding sgRNA in human HepG2 cells (89 ± 1.6%; Supplementary Fig. 2f–h), and, because the target sequence is perfectly conserved to NHPs (Supplementary Fig. 2b), we selected sgRNA_mP01 and sgRNA_hP01 for our in vivo experiments in mice and cynomolgus macaques, respectively (Fig. 1a).

In recent years, ABEs with different TadA and Cas9 variants have been established^25^. To first identify an efficient ABE variant for editing our target locus in the liver, we delivered different *Sp*Cas9-TadA constructs together with sgRNA_mP01 to the liver of 5-week-old C57BL/6J mice using a dual adeno-associated virus (AAV) intein-split system^26^ (Fig. 1b and Supplementary Fig. 3a). Six weeks after AAV-based ABE delivery, genomic DNA was isolated from the liver, and the target locus was analyzed by targeted amplicon deep sequencing. The highest editing rates (60 ± 18%; Fig. 1c) were obtained with ABEmax, in which laboratory-evolved TadA7.10 is N-terminally fused to nickase (n)*Sp*Cas9. Phenotypic analysis of these mice also revealed a significant decrease in plasma Pcsk9 levels (from 365 ± 38 ng ml^−1^ to 53 ± 9 ng ml^−1^) as well as plasma LDL levels (from 1.53 ± 0.14 mmol L^−1^ to 0.46 ± 0.09 mmol L^−1^) (Fig. 1d,e). Mice treated with TadA N-terminally fused to nuclease dead (d)*Sp*Cas9 obtained less editing (21.5 ± 5.1%; Fig. 1c), and exchanging TadA7.10 with the hyperactive TadA8e^27^ increased editing rates on the bystander adenine, but not editing of the target adenine, in the splice donor site (Fig. 1c and Supplementary Fig. 3d). In addition, identified insertion/deletion (indel) mutations at the target site were lower in ABEmax-treated versus ABE8e-treated mice (0.027 ± 0.015% versus 0.276 ± 0.057%; Fig. 1c). Together, these findings prompted us to continue all further in vivo experiments in this study with ABEmax.

### Transient base editing by LNP-mediated mRNA/sgRNA delivery

**Fig. 2:**
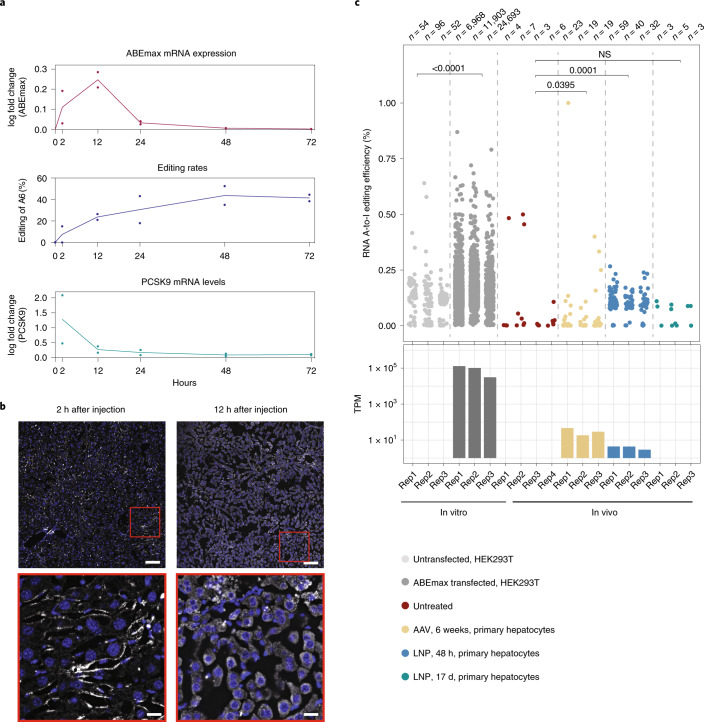
LNP-mediated ABE mRNA delivery leads to transient base editing without inducing substantial off-target deamination in the transcriptome. **a**, LNPs co-formulated with ABE mRNA and sgRNA_mP01 were systemically delivered. The top panel depicts ABEmax mRNA expression assessed by RT–qPCR. The middle panel depicts editing rates assessed by Sanger sequencing. The bottom panel depicts Pcsk9 mRNA levels assessed by RT–qPCR. The line represent mean of *n* = 2 animals per time point. **b**, Expression and localization of ABEmax mRNA 2 h or 12 h after injection of 3 mg kg^−1^ LNP assessed by smFISH in the liver. Twelve hours after injection, mRNA is predominantly cytoplasmatic. Blue, DAPI; white, ABEmax mRNA. Scale bar, top panel, 100 µm; scale bar, bottom panel, 20 µm. smFISH was performed once. **c**, Top, RNA-wide A-to-I editing assessed by whole-transcriptome sequencing. Each dot represents one editing event. The total number of editing events is indicated above. Each lane represents one individual biological replicate per animal. In vitro RNA-seq data are from HEK293T cells that were co-transfected with plasmids expressing ABEmax and sgRNA_mP01. Means of all replicates per sample were compared using one-tailed unpaired *t*-test for ex vivo samples and one-way ANOVA for in vivo samples. Bottom, telative TadA transcript expression in transcripts per million (TPM). NS, not significant.

In tissues with a slow turnover rate, AAV-mediated delivery leads to long-term transgene expression^28^ (Supplementary Fig. 3b,c). For the ‘hit-and-run’ process of genome editing, this is neither necessary nor desired, as modifications in genomic DNA are permanent. Moreover, prolonged ABE expression could lead to an accumulation of off-target mutations over time and might induce immune responses and a rejection of cells expressing bacterial Cas9 or TadA. Corroborating the latter concern, we found specific antibodies against *Sp*Cas9 and TadA in AAV-treated mice, which is in line with a recent study that detected humoral and T cell responses in mice where *Sa*Cas9 was delivered via AAV^29^ (Fig. 1f). Therefore, we next employed LNPs to transiently deliver ABEmax mRNA and sgRNA_mP01 into the mouse liver. 1-methoxyuridine-modified ABEmax mRNA was co-formulated with chemically modified sgRNAs^30,31^ in a 1:1 molecular weight ratio into LNPs (Supplementary Fig. 4a) and systemically administrated into C57BL/6J mice via tail vein injection. A single injection dose of 1 mg kg^−1^, 1.5 mg kg^−1^ and 3 mg kg^−1^ resulted in a mean editing efficiency of 3.9 ± 2.8%, 12.9 ± 5.5% and 50.9 ± 8.8%, respectively (Fig. 1g). No significant difference in editing rates was observed between the two different variants of chemically modified sgRNAs (Supplementary Fig. 4a,b). Re-dosing after 2 d in the 1.5 mg kg^−1^ and 3 mg kg^−1^ groups further increased editing rates to 39.9 ± 10% and 67.3 ± 18%, respectively, as measured from whole liver lysates (Fig. 1g and Supplementary Fig. 4c,d), and phenotypic analysis revealed substantial reduction in plasma Pcsk9 levels to 19.1 ± 4 ng µl^−1^ and plasma LDL levels to 0.64 ± 0.04 mmol L^−1^ (Fig. 1h,i and Supplementary Fig. 4e). Although LNP administration led to a temporary increase in serum transaminases, indicative of hepatocellular injury, mice were otherwise asymptomatic (Supplementary Fig. 4f,g), and neither Cas9- nor TadA-specific antibodies were detected 2.5 weeks or 15 weeks after treatment (Fig. 1j). As expected, LNPs also showed high affinity for delivery to hepatocytes; editing rates were increased to 86.9 ± 1.9% when genomic DNA was isolated from primary hepatocytes as compared to whole liver lysates, where 30% of cells are non-parenchymal^32^; in other organs, editing rates remained below 7% (testes, spleen, lung, heart, kidney, diaphragm and skeletal muscle; Supplementary Fig. 4h). As intended, we also observed that LNP-mediated mRNA/sgRNA delivery leads to transient expression and activity of the gene editing machinery: ABE mRNA levels peaked at 12 h after delivery and quickly declined thereafter; Pcsk9 mRNA levels rapidly declined within the first 24 h; and editing rates reached a plateau after 48 h (Fig. 2a,b).

### Molecular assessment of off-target editing in RNA

The generation of off-target mutations could limit clinical application of base editing. Off-target mutations can be either sgRNA dependent^11,12^ or independent of the sgRNA sequence and triggered by the promiscuous reactivity of deaminases in the mRNA or genomic DNA^13–15^. Because recent in vitro studies in human cell lines demonstrated that ABE expression causes tens of thousands of sgRNA-independent A-to-G transitions in mRNA^13,15,33^, we first assessed transcriptome-wide off-target deamination after in vivo ABE delivery using RNA sequencing (RNA-seq) (on average, 160 million reads per library). Although we were able to confirm previous in vitro studies and found that transfection of ABEmax-expressing plasmids into HEK293T cells induced, on average, more than 11,000 A-to-G transitions on the transcriptome^13,15^ (Fig. 2c and Supplementary Fig. 5a,b), neither LNP- nor AAV-mediated delivery of ABE led to a major increase in A-to-G transitions in the transcriptome of hepatocytes isolated from treated mice (on average, 44 and 20 A-to-G transitions, respectively; Fig. 2c). Moreover, off-target effects in LNP-treated animals were temporary and no longer detectable at Day 17 after injection (Fig. 2c). Previous studies suggested that unguided deamination by base editors is dose dependent^16–18^, providing a possible explanation for the low rates of off-target deamination in mRNA in vivo. Indeed, when we compared ABE expression levels in vitro and in vivo, we found that expression in transfected HEK293T cells was in the order of 3–4 magnitudes higher than in hepatocytes isolated from LNP- or AAV-treated mice (Fig. 2c).

### Molecular assessment of off-target editing in DNA

**Fig. 3:**
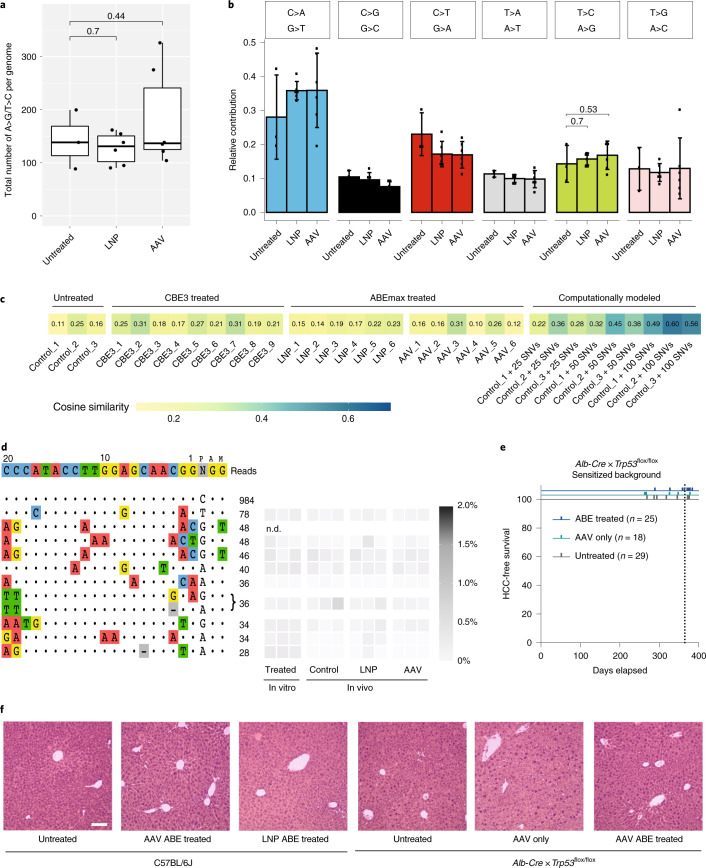
In vivo adenine base editing of *Pcsk9* does not induce substantial off-target mutations in DNA. **a**, Total number of A-to-G (including T-to-C) edits per genome. Clones from untreated control (*n* = 3), LNP-treated (*n* = 6) and AAV-treated (*n* = 6) mice. Box plots are standard Tukey plots. The centerline represents the median; the lower and upper hinges represent the first and third quartiles; and the whiskers represent ±1.5× the interquartile range. Two-tailed unpaired *t*-tests were used to compare means. **b**, Relative contributions of single-base substitutions in clones shown in **a**. Values represent mean ± s.d. Two-tailed unpaired *t*-tests were used to compare means. **c**, Heat map showing the cosine similarity of mutational signatures of control clones (untreated and CBE-treated) and ABE-treated clones (LNP and AAV) to a predetermined TadA signature^39^. 1 (match) and 0 (no similarity). For details on the clones, see Supplementary Fig. 6. In positive control clones, a TadA-specific trinucleotide mutation pattern^39^ was computationally added. **d**, sgRNA-dependent off-target sites of sgRNA-mP01 identified by CIRCLE-seq. The top ten off-target sites were analyzed by NGS in Hepa1-6 cells 5 d after transfection with plasmids encoding ABEmax and sgRNA_mP01 (in vitro) and in primary hepatocytes isolated from LNP-treated mice (3 mg kg^−1^, re-dose), AAV-treated mice (ABEmax) and untreated C57BL/6J mice. The second off-target site could not be determined (n.d.) owing to repetitiveness of the locus. Values represent the highest A-to-G conversion frequency within the protospacer. *n* = 3 biological replicates per treatment. **e**, *Alb-Cre* × *Trp53*^flox/flox^ mice treated with AAVs expressing ABEmax and sgRNA_mP01 5 weeks after birth. Depicted is the HCC-free survival of mice. *n* = number of animals per group. **f**, Histopathology of liver samples from wild-type C57BL/6J animals and *Alb-Cre* × *Trp53*^flox/flox^ animals. C57BL/6J animals untreated (*n* = 3), treated with LNP (*n* = 3) or treated with AAV (*n* = 3) were analyzed after 18 or 25 weeks, respectively. *Alb-Cre* × *Trp53*^flox/flox^ animals untreated (*n* = 28), treated with control AAV (C-terminal part of the split system and a non-targeting sgRNA) (*n* = 18) or treated with the PCSK9-targeting AAV (*n* = 25) were analyzed after 1 year. H&E staining was performed on all animals; three pictures per animal were taken. Representative images are shown. (H&E; scale bar, 50 µm).

In contrast to off-target mutations in RNA, off-target mutations in genomic DNA are permanent. Thus, the potential of ABEs to induce DNA off-target edits is an important consideration for clinical risk assessment of transient base editing approaches. Recent ex vivo studies in human induced pluripotent stem cells, two-cell stage mouse embryos and rice callus cells associated cytidine base editor (CBE) expression, but not ABE expression, with sgRNA-independent off-target deamination in genomic DNA^34–36^. To assess whether this observation holds true for in vivo adenine base editing in the liver, we analyzed the genomic DNA of hepatocytes from AAV- and LNP-treated animals by whole-genome sequencing (WGS). Considering that unguided deamination is likely to occur in a random fashion at different sites in each cell, and taking into account that all next-generation sequencing (NGS) technologies suffer from baseline error rates^37^, mutations would be overshadowed by noise if bulk DNA from a pool of cells is sequenced. Therefore, we isolated primary hepatocytes from treated animals and expanded them ex vivo^38^ to obtain enough clonal DNA for WGS at 30× coverage (Supplementary Fig. 6a). Clones without editing at the target locus were excluded, ensuring that only hepatocytes that were exposed to ABE were analyzed. Consistent with previous ex vivo studies, we found no significant increase in A > G transitions in clones from LNP- or AAV-treated mice (Fig. 3a), and, also, the relative contribution of A > G transitions to total single-nucleotide variants (SNVs) was not increased (Fig. 3b and Supplementary Fig. 6b,c). In addition, cosine similarity analysis revealed that clones derived from AAV- or LNP-treated mice showed no enrichment in TadA trinucleotide signatures^39^ compared to negative control clones (derived from untreated and CBE-treated mice) (Fig. 3c and Supplementary Fig. 6b,c).

Base editor off-target mutations in genomic DNA could also be sgRNA dependent, induced by the recruitment of the Cas9-deaminase complex to sequences similar to the target locus^11,12^. Therefore, we next investigated the target specificity of sgRNA_mP01 and performed circularization for in vitro reporting of cleavage effects by sequencing (CIRCLE-seq) and circularization for high-throughput analysis of nuclease genome-wide effects by sequencing (CHANGE-seq) analysis^40,41^ in genomic mouse DNA treated in situ with *Sp*Cas9 nuclease complexed with sgRNA_mP01 (Fig. 3d and Supplementary Fig. 7). Subsequent amplicon sequencing (>10,000 reads) of the top ten identified potential off-target sites in the liver of AAV- or LNP-treated animals showed no significant increase in A-to-G conversions or indel mutations compared to untreated control animals (Fig. 3d and Supplementary Table 1). Taken together, we found no evidence for the generation of substantial off-target mutations in genomic DNA after AAV- or LNP-mediated adenine base editing in vivo. Moreover, we did not observe malignant transformations in the livers of AAV- or LNP-treated mice after 18 and 25 weeks, and prolonged ABE expression over 1 year in mice sensitized to tumor development by liver-specific *Trp53* deletion^42,43^ did not enhance hepatocellular carcinoma (HCC) formation (Fig. 3e,f and Supplementary Fig. 8a–c). Together, these results suggest that adenine base editing in vivo is effective and safe and could potentially be used for therapeutic purposes in patients with genetic liver diseases.

### PCSK9 inhibition by adenine base editing in macaques reduces blood LDL

**Fig. 4:**
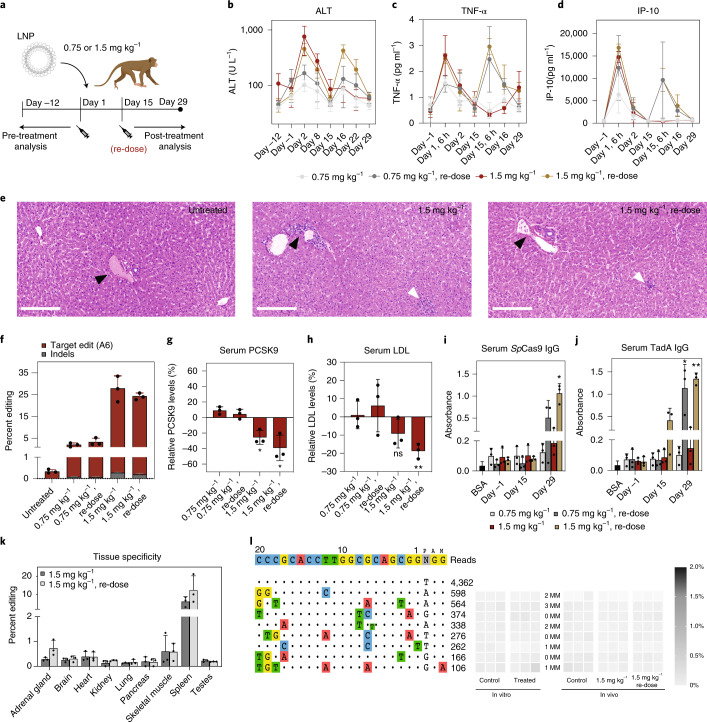
In vivo adenine base editing of the *PCSK9* locus in the liver of macaques. **a**, Schematic outline of the experiments. Levels of ALT (**b**), TNF-α (**b**) and IP-10 (**c**). Day 15 is before re-dosing; Day 15, 6 h is 6 h after re-dosing. **e**, Histopathology of liver samples from untreated, 1.5 mg kg^−1^ single-dosed and 1.5 mg kg^−1^ re-dosed animals. Three different liver lobes of all animals of the high-dose groups were examined by a trained pathologist. Only very mild lobular mixed inflammatory cell infiltration was observed (white arrowhead). Black arrowheads indicate portal tracts. H&E; scale bar, 200 μm. **f**, Percent editing in treatment groups (six liver biopsies per animal analyzed). **g**, PCSK9 levels. Serum from two time points before (Day –12 and Day −1) and after (Day 22 and Day 29) treatment was analyzed. *P = 0.020 (1.5 mg kg^−1^); *P = 0.027 (1.5 mg kg^−1^ re-dose). **h**, LDL levels. Serum from two time points before (Day –12 and Day –1) and after (Day 22 and Day 29) treatment was analyzed. NS = 0.091; **P = 0.008. **i**, Background-subtracted absorbance (*A*_450_ – *A*_540_) representing the relative amount of anti-Cas9-specific IgG antibodies. 5% BSA coating was used to determine background levels. Means were compared to Day −1. *P = 0.0095. **j**, Background-subtracted absorbance (*A*_450_ – *A*_540_) representing the relative amount of anti-TadA-specific IgG antibodies. Means were compared to Day −1. *P = 0.0336, **P = 0.0026. **k**, Editing rates in DNA isolated from other tissues than the liver. **l**, sgRNA-dependent off-target sites of sgRNA_hP01 in the human genome identified using CIRCLE-seq. The top eight hits with orthologous sites in *M. fasciularis* were analyzed by NGS in vitro (HepG2 cells transfected with plasmids encoding ABEmax and sgRNA_hP01) and in vivo (3 mg kg^−1^ single-dosed and re-dosed). Values represent the highest A-to-G conversion frequency within the protospacer. *n* = 3 biological replicates per treatment. Control: DNA isolated from untreated HepG2 cells and macaque blood cells before treatment. MM, mismatches between the human and *M. fasciularis* genome. Values represent mean ± s.d. of *n* = 3 animals. Means were compared using one-tailed paired *t*-test.

To next assess the feasibility of LNP-mediated adenine base editing in a clinically relevant large animal model, we edited *PCSK9* in adult cynomolgus macaques (*Macaca fascicularis*). Chemically modified sgRNA_hP01 (variant P1, Synthego; Supplementary Fig. 4a) was co-formulated with 1-methoxyuridine-modified ABEmax mRNA into LNP in a 1:1 weight ratio. Four groups of cynomolgus macaques, with three animals per group, received intravenous 1-h infusions of 0.75 mg kg^−1^ RNA (low dose) or 1.5 mg kg^−1^ RNA (high dose) on Day 1. Each dose level was given either as a single dose or as two doses (repeat dose) using a 2-week interval between doses (Fig. 4a). All animals were euthanized on Day 29, and tissues from different organs were collected. The administration of LNP-encapsulated ABE mRNA and sgRNA_hP01 was well tolerated in all macaques of the four dosing groups, and no animal had to be excluded during the study. Although serum transaminases (aspartate transaminase (AST) and alanine transaminase (ALT)) and a panel of pro-inflammatory cytokines and immune-modulating chemokines were transiently increased after dosing, elevated levels had resolved by 7 d and 24 h, respectively (Fig. 4b–d and Supplementary Figs. 9 and 10). No cumulative effects with repeated dosing, and no further abnormalities in blood chemistry, were observed (Supplementary Fig. 9). Moreover, a histological examination of livers at Day 29 of high-dose animals (single and repeat dose at 1.5 mg kg^−1^) indicated only sporadic and very mild lobular mixed cell infiltration (Fig. 4e). Next, we assessed on-target editing at the splice donor site of *PCSK9* intron 1. We extracted genomic DNA from postmortem biopsies of all four liver lobes (six samples per animal; Supplementary Table 4) and performed targeted amplicon deep sequencing. Although a single dose of 0.75 mg kg^−1^ led only to 2.03 ± 0.85% editing, a single dose of 1.5 mg kg^−1^ resulted in 27.6 ± 5.87% A-to-G editing of the target base, with very low indel rates (up to 0.27%; Fig. 4f). Re-dosing after 2 weeks did not further increase editing, with editing rates remaining at 3.31 ± 1.73% in the 0.75 mg kg^–1^ group and 24.14 ± 1.52% in the 1.5 mg kg^–1^ group (Fig. 4f). Notably, in high-dose animals, we observed a significant reduction in serum PCSK9 levels (26% and 39% reduction from the baseline in the single-dose group and repeat-dose group, respectively; Fig. 4g), which was associated with a lowering of serum LDL levels (9% and 19% reduction from the baseline in the single-dose group and repeat-dose group, respectively; Fig. 4h).

### Humoral immune response upon ABE treatment in macaques

In contrast to LNP-mediated adenine base editing in mice, re-dosing in macaques did not lead to a further increase in editing (Fig. 4f). A possible explanation for this finding is that the stimulation of a pro-inflammatory environment together with the expression of bacterial ABE components in the liver evoked a host response during initial treatment. This prompted us to test the serum of treated macaques for *Sp*Cas9- and TadA-specific antibodies by enzyme-linked immunosorbent assay (ELISA). When we first analyzed the 0.75 mg kg^−1^ and 1.5 mg kg^−1^ single-dose groups, we observed only a minor and statistically not significant increase in absorbance (*A*_450_ – *A*_540_) at Day 29 compared to pre-treatment (Day −1; Fig. 4i,j). However, in both repeat-dose groups, we detected *Sp*Cas9- and TadA-specific IgG antibodies at Day 29 (Fig. 4i,j). Given the high concordance between cellular and humoral immunoreactivity^44^, we speculate that LNP-treated animals also developed an ABE-specific T cell response, which might have cleared transfected hepatocytes during re-dosing. If this hypothesis is true, earlier re-dosing before the formation of adaptive immunity or transient immunosuppression might further increase editing rates.

### Off-target editing analysis in macaques

High tissue tropism of the delivery vector and high target specificity of the sgRNA are essential criteria for the safety of genome editing therapies. To assess hepatotropism of the applied LNP formulation, we analyzed on-target editing rates in nine different organs from animals of the high-dose groups. We found that editing rates in testes, brain, skeletal muscle, pancreas, lung, kidney, heart and adrenal gland remained below 1%. Only in the spleen we observed editing rates of 6.1 ± 2.7% and 12.4 ± 7.8% in single- and repeat-dose treated animals, respectively (Fig. 4k). These results are in line with a recent study that demonstrated efficient internalization of LNP-mRNA complexes in macaques by antigen-presenting cells^45^. To next address target specificity of sgRNA_hP01, we identified candidate off-target sites in the human genome. We performed CIRCLE-seq and CHANGE-seq^35,36^ on human genomic DNA isolated from HEK293T cells treated in situ with *Sp*Cas9 nuclease and sgRNA_hP01 and iGUIDE^46^ on HEK293T cells transfected with *Sp*Cas9 and sgRNA_hP01 (Fig. 4l, Supplementary Fig. 11a–c and Supplementary Table 2). To also identify candidate off-target sites specific to the genome of *M. fascicularis*, we additionally performed CHANGE-seq with sgRNA_hP01 on genomic DNA isolated from untreated macaques (Supplementary Fig. 11d). The top candidate off-target sites in the human and macaque genome were then analyzed by deep sequencing in human HepG2 cells transfected with plasmids expressing ABEmax and sgRNA_hP01 and in macaques of the high-dose groups. Despite 89% on-target editing in transfected HepG2 cells, we did not observe editing above background in any of the eight off-target candidate sites (Fig. 4l). Likewise, in none of the top candidate off-target sites in treated macaques was substantial A-to-G editing observed (Fig. 4l and Supplementary Fig. 11d).

## Discussion

Base editors are widely applied genome editing tools that hold great potential for therapeutic applications. They enable correction of all four transition mutations, which account for approximately 30% of annotated pathogenic mutations. Although protospacer adjacent motif (PAM) availability restricts the targeting scope of classical *Sp*Cas9 base editors, this limitation can theoretically be circumvented by the use of base editors that are built on novel Cas variants, making the vast majority of pathogenic transition mutations targetable^25^. Supporting this hypothesis, a recent study reported correction of over 3,000 disease-associated SNVs with more than 90% precision using different base editor variants^47^. In this study, we applied base editing to install a splice site mutation in *PCSK9,* and we show here that LNP-mediated delivery of ABE-encoding nucleoside-modified mRNA, together with chemically modified sgRNA, results in editing rates of up to 30% in the liver of macaques. These efficiencies should be sufficient for therapeutic application in several genetic liver diseases, including urea cycle disorders, phenylketonuria and tyrosinemia^48–50^. Moreover, it is conceivable that adjustment of dose levels and schedules could further increase editing rates in macaques to those observed in mice, making the PCSK9 base editing approach reported here also suitable for treating patients with familial hypercholesterolemia.

One of the major considerations for the clinical development of safe and effective genome editing therapies is the minimization or elimination of off-target editing and mutations. Thus, a therapeutically viable approach should maximize on-target editing but minimize off-target editing. We demonstrate that transient LNP-mediated ABE delivery into mouse livers enables up to 88% on-target editing in hepatocytes without inducing substantial sgRNA-dependent or sgRNA-independent (unguided) off-target editing in genomic DNA. Notably, the number of hepatocytes analyzed for unguided off-target deamination in our study was limited, and generation of mutations below our detection limit or in a sub-pool of hepatocytes cannot be excluded. Additional long-term studies in relevant model systems would, therefore, be highly valuable to further assess safety of in vivo adenine base editing. However, it is likely that the risks resulting from off-target editing are relatively low. First, over an average human lifespan, every hepatocyte collects, on average, more than 1,000 spontaneous SNVs^51^, suggesting that humans can be well capable of tolerating a large number of point mutations. Second, continuous expression of ABEmax over the period of 1 year in *Trp53* mutant mice did not increase HCC formation.

In conclusion, we provide preclinical data in mice and NHPs for the application of adenine base editing to treat genetic liver diseases. We demonstrate therapeutically beneficial editing using transient and non-viral delivery vectors without induction of considerable off-target mutations.

The remaining risks of such clinically viable base editor therapies must be carefully weighed against their benefits. This makes genetic liver diseases, which at present can be cured only by organ transplantation, ideal candidates.

## Methods

### Cell culture, transfection and harvest

Hepa1-6 (ATCC CRL-18.30) and HEK293T (ATCC CRL-321) cells were maintained in DMEM plus GlutaMAX (Thermo Fisher Scientific), and HepG2 (ATCC HB-8065) cells were maintained in EMEM (Gibco). The media were supplemented with 10% (vol/vol) FBS and 1× penicillin–streptomycin (Thermo Fisher Scientific). Cells were maintained at 37 °C and 5% CO_2_ at a confluency below 90% and seeded on 48-well cell culture plates (Greiner) for transfection. Then, 12–16 h after seeding, at approximately 70% confluency, cells were transfected using 1.5 µl of Lipofectamine 2000 (Thermo Fisher Scientific) with 750 ng of base editor plasmid (Addgene no. 112101) and 250 ng of sgRNA plasmid (Addgene no. 52963). HepG2 cells were transfected using the Neon Transfection System (Invitrogen) following the manufacturer’s instructions. Briefly, 1.2 × 10^6^ cells and 2.4 μg of the ABEmax plasmid DNA (MHp27) and 0.9 μg of the hPCSK9-gRNA (gRNA-6) plasmid DNA were resuspended in electroporation buffer R (Invitrogen) and electroporated with the following program: 1,230 V, 20 ms and three pulses. Transfection efficiency was checked by microscopy of GFP-positive cells and enriched by puromycin selection (2.5 µg ml^−1^). Cells were expanded until they reached confluency in a six-well plate. Upon detachment using TrypLE Express Enzyme (Thermo Fisher Scientific) for Hepa1-6 and HEK293T cells or Trypsin-EDTA for HepG2 cells, the cells were washed 2× in PBS and distributed for DNA lysis, RNA isolation or protein harvest.

### Genomic DNA amplification, Sanger sequencing and BEAT analysis

Next, 30 µl of a cell suspension in PBS was directly lysed using 10 µl of 4× lysis buffer (10 mM Tris-HCl at pH 8, 2% Triton X-100, 1 mM EDTA, 1% freshly added proteinase K) and incubated at 60 °C for 60 min and 95 °C for 10 min. Target sites were amplified by polymerase chain reaction (PCR) using GoTaq G2 Hot Start Green Master Mix (Promega) and the respective primer pair (Supplementary Table 5). Amplification products were purified using Agencourt AMPure XP beads (Beckman Coulter) and sequenced with the respective in-sequence primers (Supplementary Table 5) via the Sanger method. Editing efficiency was determined by BEAT analysis^52^.

### RNA isolation and RT–qPCR

RNA isolation was performed using the RNeasy Kit (Qiagen). cDNA was reverse transcribed using the GoScript Reverse Transcriptase Kit (Promega). RT–PCR was performed using GoTaq qPCR Master Mix (Promega) with specific primers for mouse or human PCSK9 and mouse B2M or human β-actin as housekeeping genes (Supplementary Table 5) and analyzed by QuantStudio 5 Real-Time PCR System (Thermo Fisher Scientific) or 7900HT Fast Real-Time PCR System (Applied Biosystems). Fold changes were calculated using the ΔΔCT method.

### Western blot

Harvested Hepa1-6 cells were lysed in RIPA buffer containing protease inhibitor and PhosSTOP phosphatase inhibitor (Sigma-Aldrich). Protein amount was determined with the Pierce BCA Protein Assay Kit (Thermo Fisher Scientific), and equal amounts of proteins were separated by SDS–PAGE electrophoresis followed by transfer to nitrocellulose membrane (Sigma-Aldrich). Membranes were incubated with goat anti-mouse-Pcsk9 (1:10,000, cat. no. AF3985-SP, R&D Systems) and rabbit anti-β-actin (1:3,000, cat. no. 4970S, Cell Signaling) or rabbit anti-GAPDH (1:5,000, cat. no. 4970, Abcam). HRP- or IRDye-conjugated secondary antibodies (donkey anti-goat: LI-COR cat. no. 926-32214; anti-rabbit: LI-COR cat. no. 926-68073; Cell Signaling cat. no. 7074; Promega cat. no. V8051) were used, and signal was revealed by enhanced chemiluminescence substrate (Thermo Fisher Scientific) or fluorescence using LI-COR.

### ELISAs

Human and NHP PCSK9 levels were determined using the Human Proprotein Convertase 9/PCSK9 Quantikine ELISA Kit (R&D Systems, cat. no. DPC900), and mouse Pcsk9 levels were determined by using the Mouse Proprotein Convertase 9/PCSK9 Quantikine ELISA Kit (R&D Systems, cat. no. MPC900) according to the manufacturer’s instructions. Anti-Cas9- or anti-TadA-specific antibodies were determined by an in-house set-up direct ELISA. In short, 10 ng of Cas9 or TadA were immobilized on 96-well polystyrene MaxiSorp plates (Thermo Fisher Scientific, cat. no. 439454) diluted in 1× ELISA Coating Buffer (BioRad, cat. no. BUF030A) for 2 h at room temperature. After washing in 1× ELISA Wash Buffer (Bio-Rad, cat. no. BUF031C), the wells were blocked for 30 min in ELISA BSA blocking solution (Bio-Rad, cat. no. BUF032C). For Cas9 detection, mouse-anti-Cas9 mAB (7A9-3A3; clone no. 14697T, Cell Signaling, cat. no. 14697) was used as positive control and standard curve. Plasma samples were diluted 1:20,000 for mouse plasma and 1:2,000 for NHP serum in Tris-buffered saline with Tween (TBS-T) and incubated for 2 h at room temperature. Goat-anti-mouse (SouthernBiotech, cat. no. 1030-05) or mouse-anti-monkey (SouthernBiotech, cat. no. 4700-05) HRP-linked secondary antibodies were used to detected protein-binding antibodies and developed using 1-Step Turbo TMB-ELISA Substrate Solution (Thermo Fisher Scientific, cat. no. 34022) and stopped after 20 min with Stop Solution for TMB Substrates (Thermo Fisher Scientific, cat. no. N301). Absorbance was measured at 450 nm and background at 540 nm; the latter was subtracted for quantification. For further background control, a 5% BSA coating was analyzed simultaneously.

### Protein production

His_6_-MBP-tev-TadA-tadA* was expressed overnight at 18 °C in *Escherichia coli* Rosetta 2 (DE3) (Novagen) cells upon induction of T7 RNA polymerase with IPTG. Cells were resuspended and lysed in 20 mM HEPES-KOH pH 7.5, 200 mM KCl, 10 mM imidazole and supplemented with protease inhibitors, using a Maximator High Pressure Homogenizer Type HPL6. Clarified lysate was loaded on a 15-ml Ni-NTA Superflow column (Qiagen) and eluted with 20 mM HEPES-KOH pH 7.5, 200 mM KCl, 200 mM imidazole. In the second step, TadA is further purified using a gradient elution from a 10-ml HiTrap Heparin HP column (GE Healthcare) equilibrated in 20 mM HEPES-KOH pH 7.5, 100 mM KCl, 1 mM DTT. Protein containing fractions are pooled, and affinity tag is removed using TEV protease with incubation overnight at 4 °C. Uncleaved TadA is removed using reverse nickel-affinity chromatography step, and the untagged TadA flowthrough is applied to a Superdex 200 16/600 column (GE Healthcare) and eluted in 20 mM HEPES-KOH pH 7.5, 200 mM KCl. Purified fractions were concentrated, flash frozen in liquid nitrogen and stored at −80 °C.

### Cloning

The sequences of the AAV constructs used in this work were generated by using pLV302 and pLV312.3 (Addgene plasmid nos.119943 and 119944) where regions of interest were exchanged using NEBuilder HiFi DNA Assembly Master Mix (NEB no. E2621). Amino acid sequences are listed in Supplementary Note 1. PCR was performed using Q5 High-Fidelity DNA Polymerase (New England Biolabs). pCMV_ABEmax_P2A_GFP was a gift from David Liu (Addgene plasmid no. 112101). lentiGuide-Puro was a gift from Feng Zhang (Addgene plasmid no. 52963). The coding sequence of ABEmax was cloned into the mRNA production plasmid behind a T7 promoter for mRNA production and into pET His6 LIC cloning vector (2Bc-T, Addgene plasmid no. 37236) for protein production.

### AAV vector production

All pseudotyped AAV8 vectors were produced by the Viral Vector Facility of the Neuroscience Center Zurich. AAV vectors were ultracentrifuged and diafiltered. Physical titers (vector genomes ml^–1^) were determined using a Qubit 3.0 Fluorometer. Identity of the packaged genomes of each AAV vector was confirmed by Sanger DNA sequencing.

### Animal studies

Mouse experiments were performed in accordance with protocols approved by the Kantonales Veterinäramt Zürich. Mice were housed in a pathogen-free animal facility at the Institute of Molecular Health Sciences at ETH Zurich and kept in a temperature- and humidity-controlled room on a 12-h light/dark cycle. For long-term studies of mice with sensitized background, conditional *Trp53*^F2-10/F2-10^ knockout mice^53^ were mated with albumin (*Alb*)-*Cre* transgenic mice^54^. Mice were fasted for 3–4 h before blood was collected from the inferior vena cava before liver perfusion. Mice were injected with 1–3 mg kg^−1^ of total RNA (LNP) or 1 × 10^12^ AAV vector genomes per mouse at 5 weeks of age. Injection volumes were 120–150 µl. Only male C57BL/6J animals were used. Male and female *Alb-Cre* × *Trp53*^flox/flox^ animals were used (untreated group: 17 males and 12 females; AAV-only group: 16 males and two females; AAV-treated group: 16 males and nine females). Studies involving NHPs were conducted at a facility accredited by the Association for Assessment and Accreditation of Laboratory Animal Care International, operating in accordance with the principles of the U.S. Food and Drug Administration’s Good Laboratory Practice and the *Guide for the Care and Use of Laboratory Animals* from the Institute of Laboratory Animal Resources (2011). All protocols were reviewed and approved by the Acuitas animal care and use committee. Male *M. fascicularis* (approximately 2 years of age) were housed in a temperature- and humidity-controlled room on a 12-h light/dark cycle. Animals received a 60-min intravenous infusion of 0.75 mg kg^−1^ or 1.5 mg kg^−1^ of total RNA, formulated in LNP and diluted in 0.9% sodium chloride USP. A volume of 5 ml kg^−1^ was administered by a 1-h infusion via the cephalic vein. Animals were fasted for 4 h before serum collection for ELISA and clinical chemistry.

### RNA synthesis and LNP encapsulation

Heavily modified sgRNA (P1) for mouse studies was synthesized using Synthego’s CRISPRevolution platform using solid-phase phosphoramidite chemistry. After synthesis and a series of post-processing steps and purification, oligonucleotides were quantified by ultraviolet (UV) absorption, and their identity and quality were confirmed using an Agilent 1290 Infinity II liquid chromatography system coupled with an Agilent 6530B Quadrupole time-of-flight mass spectrometry (Agilent Technologies) in a negative ion polarity mode. Chemically ultra-heavily modified sgRNAs (P2) were ordered from Agilent Technologies. Heavily modified sgRNAs for large-scale production for NHP studies were synthesized using an ÄKTA Oligopilot Plus 100 oligonucleotide synthesizer at a 112-µmol scale. After synthesis and de-protection steps, the oligo was subjected to solid-phase extraction using an ÄKTA Explorer FPLC system. This material then underwent further purification and quality assessment using the Agilent 1200 HPLC System. HPLC fractions were selected, combined and processed by tangential flow filtration using the Pall Minimate EVO TFF system. Final product quantity was evaluated using UV absorption, and its identity and quality were confirmed using Agilent 1290 Infinity II liquid chromatography system coupled with an Agilent 6530B Quadrupole time-of-flight mass spectrometry (Agilent Technologies) in a negative ion polarity mode.

The coding sequence of ABEmax was cloned into the mRNA production plasmid. mRNA production and LNP encapsulation were performed as described^55^. Briefly, mRNAs were transcribed to contain 101 nucleotide-long poly(A) tails. m1Ψ-5′-triphosphate (TriLink no. N-1081) instead of UTP was used to generate modified nucleoside-containing mRNA. Capping of the in vitro transcribed mRNAs was performed co-transcriptionally using the trinucleotide cap1 analog, CleanCap (TriLink, no. N-7413). mRNA was purified by cellulose (Sigma-Aldrich, no. 11363–250G) purification as described^56^. All mRNAs were analyzed by agarose gel electrophoresis and were stored frozen at −20 °C. Cellulose-purified m1Ψ-containing mRNA, together with the synthesized sgRNA, were encapsulated in LNPs. LNPs were formulated as described previously^57^. In short, an ethanolic solution of 1,2-distearoyl-*sn*-glycero-3-phosphocholine, cholesterol, a PEG lipid and an ionizable cationic lipid was rapidly mixed with an aqueous solution (pH 4) containing SpCas9-ABEmax mRNA and sgRNA (1:1 weight ratio) using an in-line mixer. The ionizable lipid and LNP composition are described in U.S. patent application US 2016/0376224 A1 (2016), with the ionizable lipid (pKa in the 6.0–6.5 range) belonging to lipid class defined by the structure shown in Supplementary Fig. 4a. The resulting LNP formulation was dialyzed overnight against 1× PBS, 0.2-μm sterile filtered and stored at −80 °C at a concentration of 1 μg μl^−1^ of total RNA. LNP had an average hydrodynamic diameter of 67–71 nm with a polydispersity index of 0.02–0.06 as determined by dynamic light scattering (Malvern Nano ZS Zetasizer) and a mode size of 67–75 nm as determined by nanoparticle tracking analysis (Malvern Panalytical NanoSight NS300). Encapsulation efficiencies of SpCas9-ABEmax mRNA and sgRNA in the LNP were both at 96% measured by the Quant-iT Ribogreen Assay (Life Technologies). Acuitas will provide the LNP used in this work to any academic investigator who would like to test it.

### Primary hepatocyte isolation

Mice were euthanized using CO_2_ and immediately perfused with Hank’s balanced salt solution (Thermo Fisher Scientific) plus 0.5 mM EDTA via the inferior vena cava and a subsequent incision in the portal vein. During this step, one liver lobe was squeezed off via a thread to inhibit perfusion of this lobe to collect whole liver samples for embedding and whole liver lysates. After blanching of the liver, mice were perfused with digestion medium (low-glucose DMEM plus 1× penicillin–streptomycin (Thermo Fisher Scientific), 15 mM HEPES and freshly added Liberase (Roche)) for 5 min. Livers were isolated in cold isolation medium (low-glucose DMEM supplemented with 10% (vol/vol) FBS plus 1× penicillin–streptomycin (Thermo Fisher Scientific) and GlutaMax (Thermo Fisher Scientific)), and the liver was gently dissociated to yield a cell suspension that was passed through a 100-µm filter. The suspension was then centrifuged at 50*g* for 2 min and washed with isolation medium 2–3 times until the supernatant was clear. The primary hepatocytes were pelleted for DNA or RNA isolation.

### Genomic DNA isolation and HTS

Genomic DNA from mouse tissues was isolated using the DNeasy Blood and Tissue Kit (Qiagen) according to the manufacturer’s protocol or directly lysed using direct lysis buffer: 10 µl of 4× lysis buffer (10 mM Tris-HCl pH 8, 2% Triton X-100, 1 mM EDTA, 1% freshly added proteinase K) and incubated at 60 °C for 60 min and 95 °C for 10 min. Target sites were amplified by PCR using GoTaq G2 Hot Start Green Master Mix (Promega) or NEBNext High-Fidelity 2× PCR Master Mix and the respective primer pair (Supplementary Table 6) in 26 cycles. The PCR product was purified using 0.8× Agencourt AMPure XP beads (Beckman Coulter) and amplified with primers containing sequencing adaptors for another six cycles. The products were gel purified and quantified using the Qubit 3.0 fluorometer with the dsDNA HS Assay Kit (Thermo Fisher Scientific). Samples were sequenced on Illumina MiSeq. After demultiplexing, the samples were analyzed using CRISPResso2 (ref. ^58^).

### Clinical chemistry and cyokine and inflammatory biomarkers

Total cholesterol, triglyceride, high-density lipoprotein (HDL), AST and ALT levels from all mouse samples were measured as routine parameters at the Division of Clinical Chemistry and Biochemistry at the University Children’s Hospital Zurich using Alinity ci-series. LDL levels were calculated by using the Friedewald formula. NHP serum was subjected to a full clinical chemistry panel, including ALT, AST, total bilirubin, LDL cholesterol, HDL cholesterol and total cholesterol. Approximately 1 ml of blood was taken from the femoral vein, processed to serum and analyzed using a Beckman Coulter analyzer. For cytokine and inflammatory biomarker analyses, approximately 0.8 ml of blood was processed to serum, and a panel of ten cytokine and inflammatory biomarkers (IFN-α2a, IL-18, IL-1RA, IL-1β, IL-6, IP-10, MCP-1, MIP-1α, MIP-1β and TNF-α) was evaluated using U-PLEX Biomarker Group 1 (NHP) assay kits (Meso Scale Diagnostics).

### Tissue cryosections

Mouse livers were perfused with Hank’s buffer and bound off before further perfusion. The separated section was fixed in 4% paraformaldehyde (PFA) at 4 °C overnight. Tissues were transferred to a 30% sucrose solution overnight at 4 °C and embedded in OCT compound in cryomolds (Tissue-Tek). Frozen tissues were sectioned at 7 µm at −20 °C, and mounted directly on Superfrost Plus slides (Thermo Fisher Scientific). Cryosections were counterstained with DAPI (Thermo Fisher Scientific) and mounted in VECTASHIELD mounting medium (Vector Labs). Two frozen sections were analyzed per mouse per tissue.

### Single-molecule fluorescence in situ hybridization

The ABEmax probe library was designed using Stellaris FISH Probe Designer Software (Biosearch Technologies) (Supplementary Table 7) and coupled to Cy5 as described^59^. Livers were fixed in 4% PFA in PBS for 3 h and subsequently agitated in 30% sucrose and 4% PFA in PBS overnight at 4 °C. Fixed tissues were embedded in Tissue-Tek OCT Compound (Sakura, 4583). Then 8-μm-thick sections were sectioned onto poly-l-lysine-coated coverslips, air dried for 5 min, fixed for 15 min in 4% PFA and permeabilized overnight in 70% EtOH. The liver sections were hybridized with single-molecule fluorescence in situ hybridization (smFISH) probe sets according to a previously published protocol^60^. DAPI (Sigma-Aldrich) was used as nuclear counterstain. smFISH imaging was performed on a Leica THUNDER 3D live cell imaging system, using the following THUNDER computational clearing settings: Feature Scale (nm): 350; Strength (%): 98; Deconvolution settings: Auto; and Optimization: High.

### Histology and staining

Tissues were fixed using 4% PFA at 4 °C overnight and dehydrated the next day before paraffinization. Paraffin blocks were cut into 5-μm-thick sections, deparaffinized with xylene and rehydrated. Sections were stained for hematoxylin and eosin (H&E) or Sirius Red and examined for histopathological changes.

### Microscopy

Mouse tissue was imaged using a Zeiss Apotome. Imaging conditions and intensity scales were matched for all images. Images were taken using Zeiss software Zen2 and analyzed by Fiji ImageJ software (v1.51n)^61^.

### Guide-dependent off-target prediction and analysis

For CIRCLE-seq and CHANGE-seq, the sgRNA was first tested for functionality by digesting the Sanger amplicon described above. The library was prepared as previously described^40,41^. Data were processed using version 1.1 of the CIRCLE-seq analysis pipeline (https://github.com/tsailabSJ/circleseq) with the following parameters: ‘window_size: 3; mapq_threshold: 50; start_threshold: 1; gap_threshold: 3; mismatch_threshold: 6; merged_analysis: True, variant_analysis: True’. The respective target sites were deep sequenced and covered by at least 10,000 reads per site. Highly repetitive sequences were further processed by extracting the amplicon with cutadapt^62^ (v3.1) excluding the protospacer region. If this was also not possible because the region was too similar to a different site in the genome, the off-target editing events could not be determined. For iGUIDE, libraries were prepared as previously described^46^.

### RNA-seq experiments and data analysis

RNA library preparation was performed using the TruSeq Stranded Total RNA Kit (Illumina) with ribosomal RNA (rRNA) deletion. RNA-seq libraries were sequenced on an Illumina NovaSeq machine at the Functional Genomics Center Zurich, achieving an average of more than 160 million paired-end (PE) reads per sample. Quality control, pre-processing, alignment of RNA-seq reads: Quality of Illumina PE RNA-seq reads was evaluated using FastQC version 0.11.7 (https://www.bioinformatics.babraham.ac.uk/projects/fastqc/). Using FastQ Screen version 0.11.1 (https://www.bioinformatics.babraham.ac.uk/projects/fastq_screen/), potential sample contaminations (genomic DNA, rRNA and mycoplasma) were screened against a custom database including UniVec (https://www.ncbi.nlm.nih.gov/tools/vecscreen/univec/), RefSeq mRNA sequences, selected genome sequences (human, mouse, arabidopsis, bacteria, virus, phix, lambda and mycoplasma) (https://www.ncbi.nlm.nih.gov/refseq/) and SILVA rRNA sequences (https://www.arb-silva.de/). Illumina PE reads were pre-processed using fastp version 0.20.0 to trim off sequencing adaptors and low-quality ends (average quality lower than 20 within a 4-nt window). Flexbar version 3.0.3 was used to remove the first six bases of each read, which showed priming bias introduced by the library preparation protocol^63^. PE reads longer than 50 bp were trimmed to 50 bp before being aligned to remove overlapping reads ends, which can inflate allele frequency calculation and variant calls. Quality controlled reads (average quality 20 and above, read length 20 and above) were aligned to the reference genomes (mouse reference genome: GRCm38.p5, Ensembl release 91; human reference genome: GRCh38.p10, Ensembl release 91) using STAR version 2.7.0e with two-passes mode. PCR duplicates were marked using Picard version 2.9.0. Read alignments were comprehensively evaluated in terms of different aspects of RNA-seq experiments, such as sequence quality, genomic DNA and rRNA contamination, GC/PCR/sequence bias, sequencing depth, strand specificity, coverage uniformity and read distribution over the genome annotation, using R scripts in ezRun (https://github.com/uzh/ezRun/) developed at the Functional Genomics Center Zurich. RNA sequence variant calling and filtering: Variant calling from RNA-seq reads was performed according to Genome Analysis Toolkit (GATK) Best Practices (https://gatkforums.broadinstitute.org/gatk/discussion/3891/calling-variants-in-rnaseq). In detail, GATK (v4.1.2.0) tool SplitNCigarReads was applied to post-process the read alignments. Afterwards, variants were called using HaplotypeCaller (GATK v4.1.2.0) on PCR-deduplicated, post-processed aligned reads. Variant loci in base editor overexpression experiments were filtered to exclude sites without high-confidence reference genotype calls in the control experiment. For a given SNV, the read coverage in the control experiment should be above the 90th percentile of the read coverage across all SNVs in the corresponding overexpression experiment. Only loci having at least 99% of reads containing the reference allele in the control experiment were kept. Only sites with at least ten reads in the treated sample were considered. Quantification of gene expression: Transcript expression was calculated using kallisto (v0.44.0).

### WGS and data analysis

Upon confirmation of on-target editing, DNA was harvested using the QIAamp DNA Mini Kit (Qiagen) or Quick DNA Microprep Kit (Zymo Research) according to manufacturer instructions. DNA concentrations were determined using the Qubit dsDNA HS Kit (Invitrogen). WGS was performed at a mean coverage of 30× using an Illumina NovaSeq. Read alignment, variant calling and variant filtering: Sequence reads were mapped against mouse reference genome GRCm38 by using the Burrows–Wheeler Aligner version 0.7.5 mapping tool^64^ with settings ‘bwa mem -c 100 -M’. Sequence reads were marked for duplicates by using Sambamba version 0.4.732 and realigned per donor by using the GATK IndelRealigner version 2.7.2. Raw variants were multisample-called by using the GATK HaplotypeCaller version 3.4-46 (ref. ^65^) and GATK-Queue version 3.4-46 with default settings and additional option ‘EMIT_ALL_CONFIDENT_SITES’. The quality of variant and reference positions was evaluated by using GATK VariantFiltration version 3.4-46 with options ‘-snpFilterName LowQualityDepth -snpFilterExpression “QD < 2.0” -snpFilterName MappingQuality -snpFilterExpression “MQ < 40.0” -snpFilterName StrandBias -snpFilterExpression “FS > 60.0” -snpFilterName HaplotypeScoreHigh -snpFilterExpression “HaplotypeScore > 13.0” -snpFilterName MQRankSumLow -snpFilterExpression “MQRankSum < -12.5” -snpFilterName ReadPosRankSumLow -snpFilterExpression “ReadPosRankSum < -8.0” -cluster 3 -window 35’. Full pipeline description and settings are also available at https://github.com/UMCUGenetics/IAP. To obtain high-quality somatic mutation catalogs, we applied post-processing filters as described^51^. Briefly, we considered variants at autosomal chromosomes without any evidence from a paired control sample (genomic DNA isolated from untreated tissue from the same mouse); passed by VariantFiltration with a GATK phred-scaled quality score ≥100 for base substitutions and ≥250 for indels; a base coverage of at least 20× in the clonal and paired control sample; mapping quality ≥60; and no overlap with single-nucleotide polymorphisms in the Single Nucleotide Polymorphism Database version 142. We additionally filtered base substitutions with a GATK genotype quality (GQ) score lower than 99 or 10 in clonal or paired control samples, respectively. For indels, we filtered variants with a GQ score lower than 99 in both clonal and paired control samples and filtered indels that were present within 100 bp of a called variant in the control sample. In addition, for both SNVs and indels, we considered only variants with a variant allele frequency (VAF) of 0.2 or higher in the clones to exclude in vitro accumulated mutations^51,66^. The scripts are available at https://github.com/ToolsVanBox/SNVFI and https://github.com/ToolsVanBox/INDELFI. Owing to the karyotypically unstable nature of the cells and for the fair comparison of the number of mutations in the later analysis, only the mutations from the regions considered as diploid (1.5 < ratio < 2.5 from the Control-FREEC^67^ output when the samples were treated as diploid) and callable were included. The absolute number of mutations was corrected for the lengths of the accounted genomic regions. Mutational profile and signature analysis: The numbers of six substitution types (C > A, C > G, C > T, T > A, T > C and T > G) or 96-trinucleotide mutation types (six substitution types with 5′ and 3′ flanking bases) were reported, and the frequencies of the 96-trinucleotide mutations were plotted for every mouse using an in-house-developed R package^68^. For the normalized absolute number and relative amount of six substitution types, the samples were classified based on the injected chemicals; for each group, the mean and standard deviation were calculated and plotted. To illustrate the potential TadA activity in the samples, the identified TadA motif^39^ was used as TadA signature for cosine similarity comparison. The 96-trinuclueotide frequencies were pooled from the two signatures and normalized so that the frequencies add up to 1. The 96-nt TadA signature was deduced under the assumption that other substitutions do not contribute to the TadA signature. For the three control mouse samples, the 96-nt mutational profile was constructed and normalized by the total number of SNVs and multiplied by the median number of SNVs (428 SNVs) to make them comparable between the samples. To mimic the TadA activity on the mutational profile, the additional number of SNVs were distributed over the 96-nt mutational patterns according to the determined TadA signature, for 10, 25, 50 and 100 SNVs. Any decimal values were rounded and summed to the profiles of the controls. For all the samples and the TadA signature-added controls, cosine similarity with the TadA signature was calculated using MutationalPatterns^68^ in R. To calculate the variant detection sensitivity of our method, we identified germline variants and counted how many of them were found in the clones. To exclude potential artifacts in our data, the direct output from the IAP pipeline was further filtered with the following criteria: located in diploid and CALLABLE region, passed by VariantFiltration with a GATK phred-scaled quality score ≥100, GATK GC score equals 99, base coverage of at least 20× in all the clones and the bulk samples, does not overlap with the variants in our blacklists (available upon reasonable request) and present as a heterozygous variant (VAF ≥ 0.3) in the three bulk samples. Our filtering resulted in 86 heterozygous variants, and any position with VAF < 0.3 was counted as absent in the clones. The global maximum-likelihood estimates and the confidence intervals for both mice groups were calculated using the dNdScv package^69^ and plotted using ggplot2 (ref. ^70^) in R. Called SNVs were compared among groups using the online tool by the van de Peer lab (http://bioinformatics.psb.ugent.be/webtools/Venn/) provided by the VIB/UGent. Comparison of more than six groups was analyzed and but retrospectively visualized using Adobe Illustrator.

### Statistical analyses

A priori power calculations to determine sample sizes for animal experiments were performed using G*Power^71^. Statistical analyses were performed using GraphPad Prism 8.0.0 for macOS. Sample sizes and the statistical tests used are described in the figure legends. *P* < 0.05 was considered statistically significant.

### Reporting Summary

Further information on research design is available in the Nature Research Reporting Summary linked to this article.

## Online content

Any methods, additional references, Nature Research reporting summaries, source data, extended data, supplementary information, acknowledgements, peer review information; details of author contributions and competing interests; and statements of data and code availability are available at 10.1038/s41587-021-00933-4.

## Supplementary information

Supplementary Information Supplementary Figs. 1–11, Tables 1–7, Notes 1–3 and References

Reporting Summary

## Data Availability

The main data supporting the results in this study are available in the paper and its Supplementary Information. High-throughput sequencing data are publicly available under the following accession numbers: GSE168365 (Gene Expression Omnibus datasets for targeted amplicon sequencing and RNA-seq) and PRJEB41832 (Sequence Read Archive dataset for WGS samples).

## Code availability

Scripts used to quantify on-target and off-target editing are listed in the Methods.
