# Supplementary material for: In vivo adenine base editing of PCSK9 in macaques reduces LDL cholesterol levels
Source: Nat Biotechnol. 2021 May 19;39(8):949–57. doi: 10.1038/s41587-021-00933-4 (PMC8352781; doi:10.1038/s41587-021-00933-4)
Supplement: Supplementary file 1 — Supplementary Figs. 1–11, Tables 1–7, Notes 1–3 and References [file 41587_2021_933_MOESM1_ESM.pdf]

nature portfolio

https://doi.org/10.1038/s41587-021-00933-4

Supplementary information

In vivo adenine base editing of *PCSK9* in macaques reduces LDL cholesterol levels

In the format provided by the authors and unedited

Supplementary Information:

In vivo adenine base editing of *PCSK9* in mice and macaques reduces LDL-cholesterol levels

Supplementary Figure 1 Identification of sgRNAs for introducing splice site mutations in murine *Pcsk9* by adenine base editing. 2

Supplemental Figure 2 | Adenine base editing with sgRNA-mP01 and sgRNA-hP01 in murine and human liver cell lines leads to the downregulation of PCSK9. 3

Supplementary Figure 3 | AAV-mediated delivery of different ABE variants. 4

Supplementary Figure 4 | sgRNA modification patterns and *in vivo* editing after co-formulation with ABEmax mRNA into LNP and systemic delivery. 5

Supplementary Figure 5 | A-to-I off-target editing on the transcriptome. 7

Supplementary Figure 6 | 96-nt profile plot of clonally expanded hepatocytes. 8

Supplementary Figure 7 | Detection of potential off-target sites for sgRNA-mP01 10

Supplementary Figure 8 | Adenine base editing in Alb-Cre x Trp53$^{flox/flox}$ mice. 11

Supplementary Figure 9 | Blood samples of macaques were analyzed with a clinical chemistry panel before and during ABE treatment. 12

Supplementary Figure 10 | Blood samples of macaques were analyzed with an inflammatory biomarker and cytokine panel before and during ABE treatment. 13

Supplementary Figure 11 | Detection of potential off-target sites for sgRNA-hP01 14

Supplementary Table 1 | Mouse CIRCLE-seq off-target sites analyzed by targeted amplicon sequencing 15

Supplementary Table 2 | Human CIRCLE-seq off-target sites analyzed by targeted amplicon sequencing 15

Supplementary Table 3 | Additional mouse sgRNAs tested in this study. 15

Supplementary Table 4 | On-target sequencing of DNA from liver biopsies of treated macaques 16

Supplementary Table 5 | Sanger and RT-qPCR primers used in this study. 16

Supplementary Table 6 | High throughput (HTS) sequencing primers used in this study 17

Supplementary Table 7 | smFISH probe sequences against ABEmax mRNA 18

Supplementary Note 1 | Amino Acid sequences of AAV vectors used in this study 19

Supplementary Note 2 | Javascript code for evaluation of the highest A to G conversion within the Protospacer 20

Supplementary Note 3 | Uncropped Western Blot analysis images and PCR Agarose gel images 22

Supplementary References 22

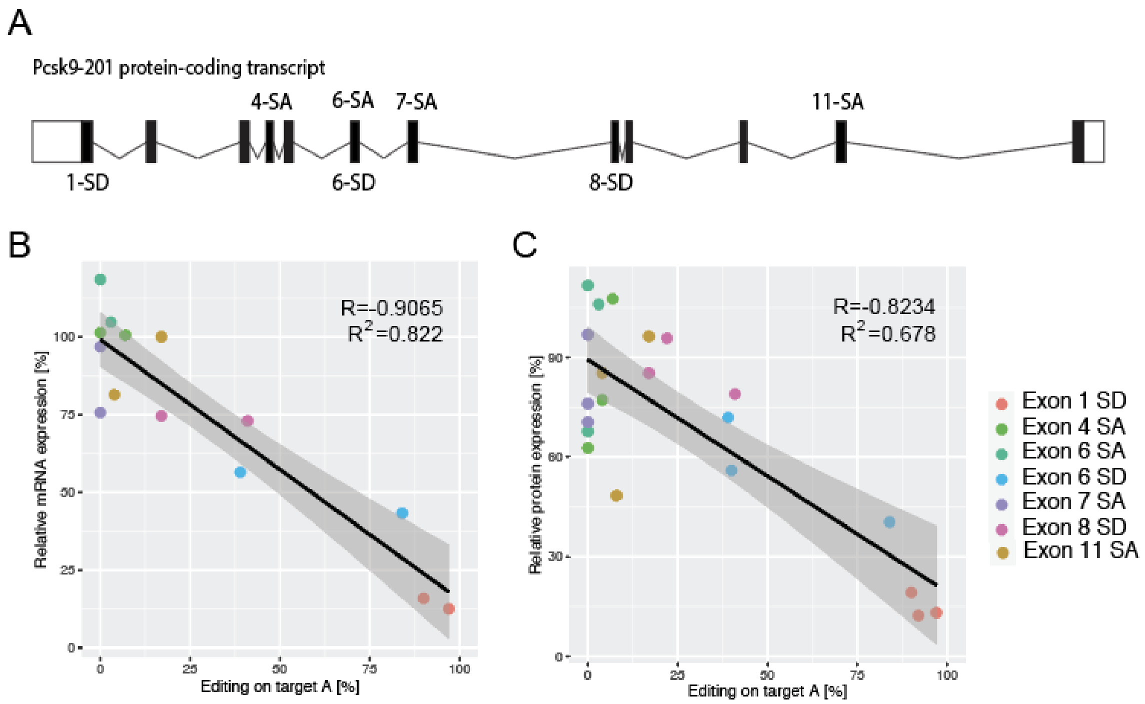

Supplemental Figure 1 | Identification of sgRNAs for introducing splice site mutations in murine *Pcsk9* by adenine base editing. (A) Overview of tested sgRNAs at their respective target locus in murine *Pcsk9*. The conserved GT motif at the beginning of an intron – canonical splice-donor (SD) sites– or the conserved AG motif at the end on an intron – splice-acceptor (SA) sites – were targeted. (B) Correlation between target A editing efficiency and *Pcsk9* mRNA levels as determined by RT-qPCR. (C) Correlation between target A editing efficiency and Pcsk9 protein levels as determined by western blot analysis. Experiments were performed in n=3 independent biological replicates. Shaded areas denote 95% confidence intervals. Hepa1-6 cells upon treatment with the indicated sgRNA. (sgRNA-1-SD = sgRNA-mP01).

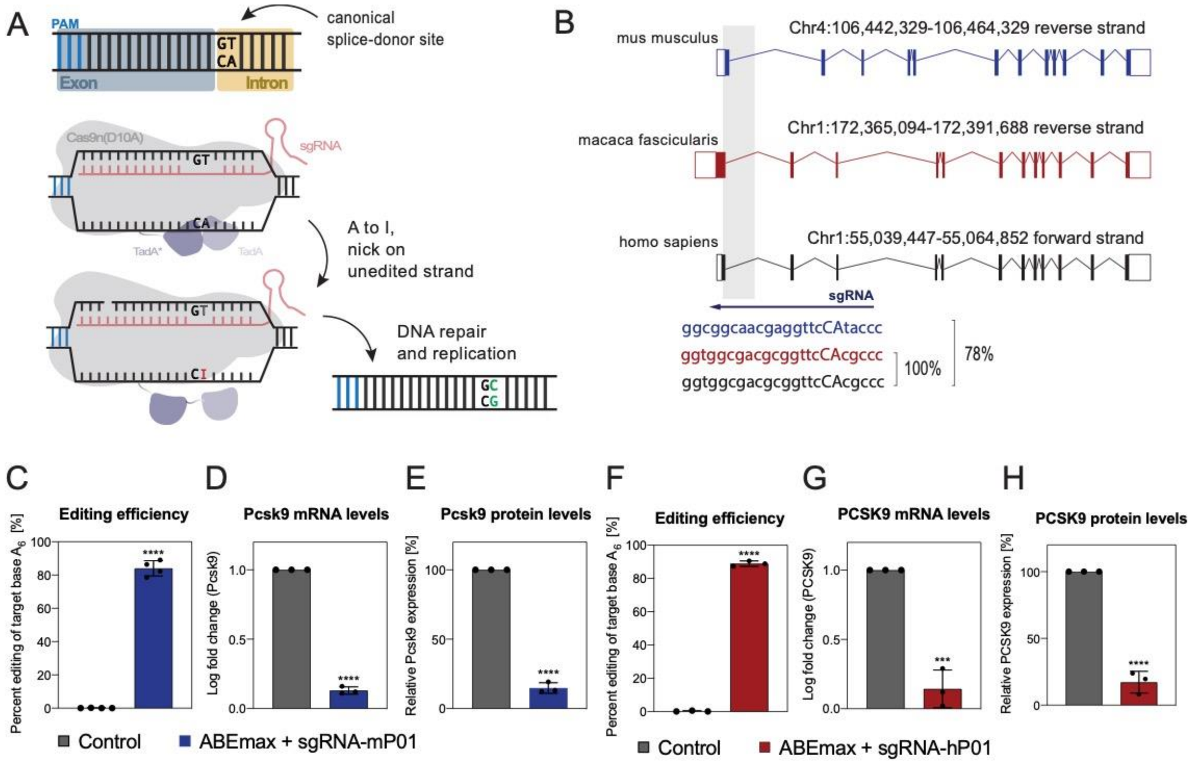

Supplementary Figure 2 | Adenine base editing with sgRNA-mP01 and sgRNA-hP01 in murine and human liver cell lines leads to the downregulation of PCSK9. (A) Schematic illustration of the adenine base editing approach for the disruption of the canonical splice-donor site of the first intron of *PCSK9*. The conserved GT motif is converted to GC by an A-to-G nucleotide conversion on the opposing strand. (B) Overview of *PCSK9* orthologs (Mus musculus, Macaca fascicularis, and Homo sapiens). Highlighted is the first splice-donor site of intron 1. The sequences of the reverse-binding sgRNAs for each ortholog is indicated below. Capital letters indicate the reverse complement bases of the targeted splice-donor site. (C) Percent editing in the mouse liver cell line Hepa1-6. Values show percent of A-to-G editing of the targeted adenine at protospacer position 6 (A6), counted from the PAM most distal base. Values represent mean ± s.d. of n=4 biologically independent experiments. (D) Log fold change of murine *Pcsk9* mRNA as determined by RT-qPCR. Values represent mean ± s.d. of n=3 biologically independent experiments. (E) Relative Pcsk9 protein expression as determined by western blotting. Pcsk9 protein expression was normalized to the housekeeping gene beta-actin. Values represent mean ± s.d. of n=3 biologically independent experiments. (F) Percent editing in the human liver cell line HepG2. Values show percent A to G editing of the targeted adenine at protospacer position 6 (A6), counted from the PAM most distal base. Values represent mean ± s.d. of n=3 biologically independent experiments. (G) Log fold change of human *PCSK9* mRNA as determined by RT-qPCR. Values represent mean ± s.d. of n=3 biologically independent experiments. ***P=0.0004 (H) Relative PCSK9 protein expression as determined by ELISA. Values represent mean ± s.d. of n=3 biologically independent experiments. Means were compared using two-tailed unpaired t-tests. *** p<0.001, **** p<0.0001

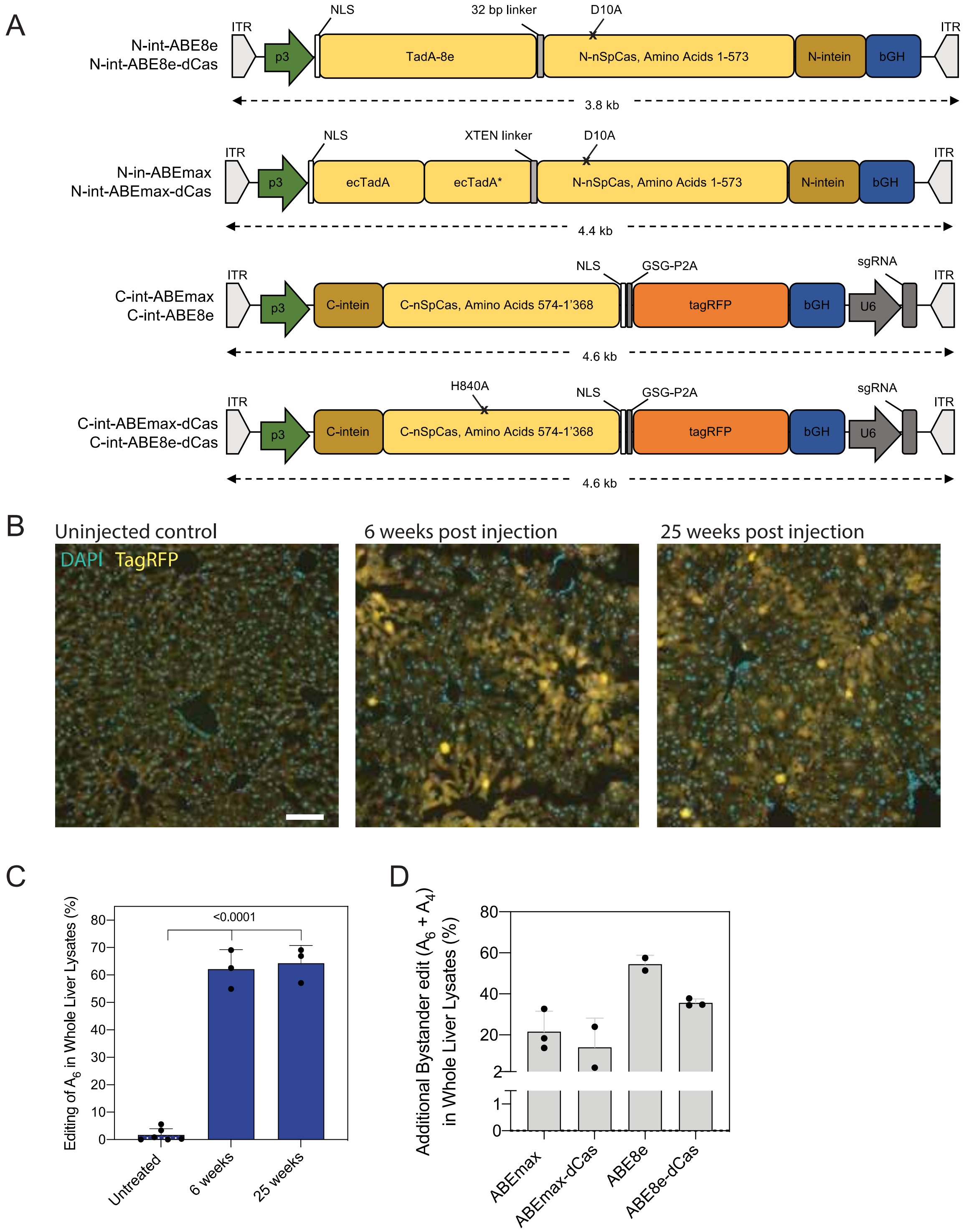

Supplementary Figure 3 | AAV-mediated delivery of different ABE variants. (A) Schematic maps of vector genomes packaged into AAV8 capsids. n*Sp*Cas9 (D10A mutation), d*Sp*Cas9 (D10A mutation and H840A mutation). ITR, internal terminal repeat; NLS, nuclear localization signal; bGH, bovine growth hormone poly(A) signal. (B) Representative cryosection of mouse livers i.v. injected with AAV8 encapsulated N-int-ABEmax + C-int-ABEmax in a 50:50 ratio. Cryosections were imaged from n = 3 animals. 3 pictures per animal were analyzed. Nuclei are stained with DAPI. Red: tagRFP expression. Scale bar, 50 µm. (C) Percent editing of target adenine editing in the protospacer of sgRNA-mP01. Values represent mean ± s.d. of n=6, n=3, n=3 biologically independent experiments. Means were compared using one-way-ANOVA. (D) Percentage of reads from total reads with additional A-to-G editing other than the target adenine (A_6) after AAV-mediated treatment with different adenine base editor (ABE) variants. Mice were injected with AAV expressing the indicated ABE variant and analyzed after 6 weeks. Values represent mean ± s.d. of n=3, n=2, n=2, n=3 biologically independent experiments.

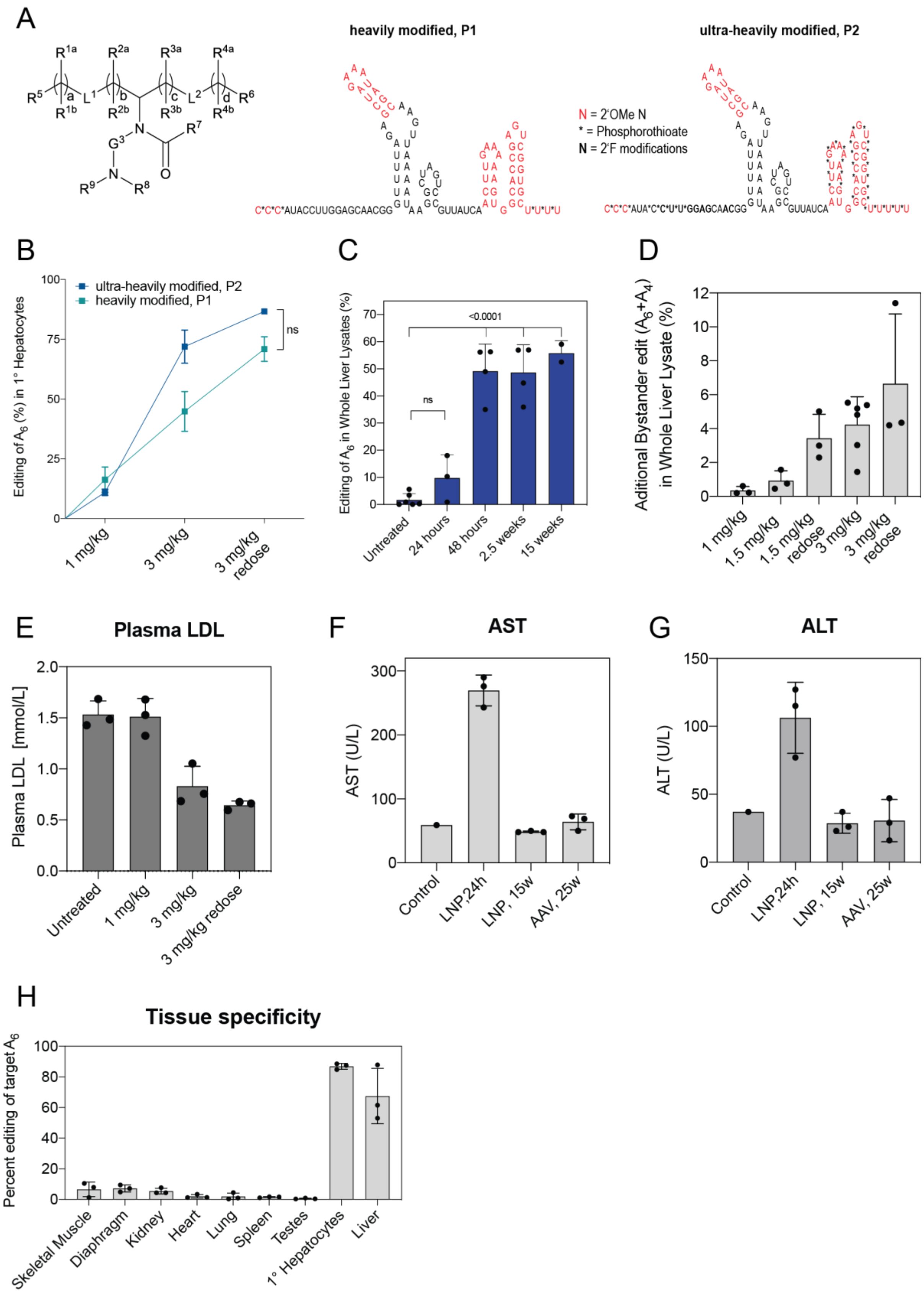

Supplementary Figure 4 | sgRNA modification patterns and *in vivo* editing after co-formulation with ABEmax mRNA into LNP and systemic delivery. (A) The left panel depicts the structure of the ionizable lipid. The ionizable lipid used in these studies belongs to the chemical class defined by the structure shown here. The L1 and L2 are each independently –O(C=O) , (C=O)O ; G3 is C1-C6 alkylene; R1a and R4a are, at each occurrence, independently H or C1-C8 alkyl; R1b R2a, R2b R3a R3b R4b are, at each occurrence, H; R5 and R6 are methyl; R7 is C6-C9 alkyl; R8 and R9 are each independently C1 alkyl; or R8 and R9, together with the

nitrogen atom to which they are attached, form a 5-membered heterocyclic ring and a, b, c and d are each independently an integer from 5 to 9. The right panel illustrates the different modification patterns tested for sgRNA_mP01. 2’ O-methyl ribonucleotides (2’OMe N) are indicated in red. 2’fluoro-ribonucleotides are indicated in bold letters. Asterisks indicate phosphorothioate bonds. The modification pattern of sgRNA_hP01 used in the macaque experiments was identical to the variant P1 of sgRNA_mP01, and was provided by Synthego. We used the P1 modification pattern for sgRNA_hP01 as it was more readily obtainable in large quantities and as it is also available in GMP-grade. (B) sgRNAs P1 and P2 were tested in different dosing regimens. No significant difference in editing efficiency was detected. ns, non-significant. Values represent mean ± s.d. of n=3 biologically independent experiments. Means were compared using a two-tailed unpaired t-test. (C) Editing efficiencies over time with LNP-encapsulated ABEmax mRNA and sgRNA_mP01 (variant P2) using a single 3 mg/kg dose over time. Values represent mean ± s.d. of n=6, n=3, n=4, n=4, n=2 biologically independent experiments. Means were compared using one-way ANOVA. ns, non-significant (D) Percentage of reads from total reads with additional A-to-G editing other than the target adenine (A_6) after LNP treatment. Values represent mean ± s.d. of n=3 biologically independent experiments. (E) Plasma LDL levels from untreated, 1 mg/kg, 3 mg/kg, and 3 mg/kg redosed C57BL/6J mice. Values represent mean ± s.d. of n=3 biologically independent experiments. (F) Plasma aspartate transaminase (AST) levels upon LNP- or AAV-mediated delivery. Values represent mean ± s.d. of n=3 biologically independent experiments. (G) Plasma alanine transaminase (AST) levels upon LNP- or AAV-mediated delivery. Values represent mean ± s.d. of n=3 biologically independent experiments. (H) Mice were systemically dosed twice at a dose of 3mg/kg. Editing efficiency of the target A was assessed from other tissues by targeted amplicon sequencing. Editing efficiency increased by 37 ± 10 % in isolated primary (1°) hepatocytes compared to whole liver lysates. Values represent mean ± s.d. of n=3 biologically independent experiments.

A

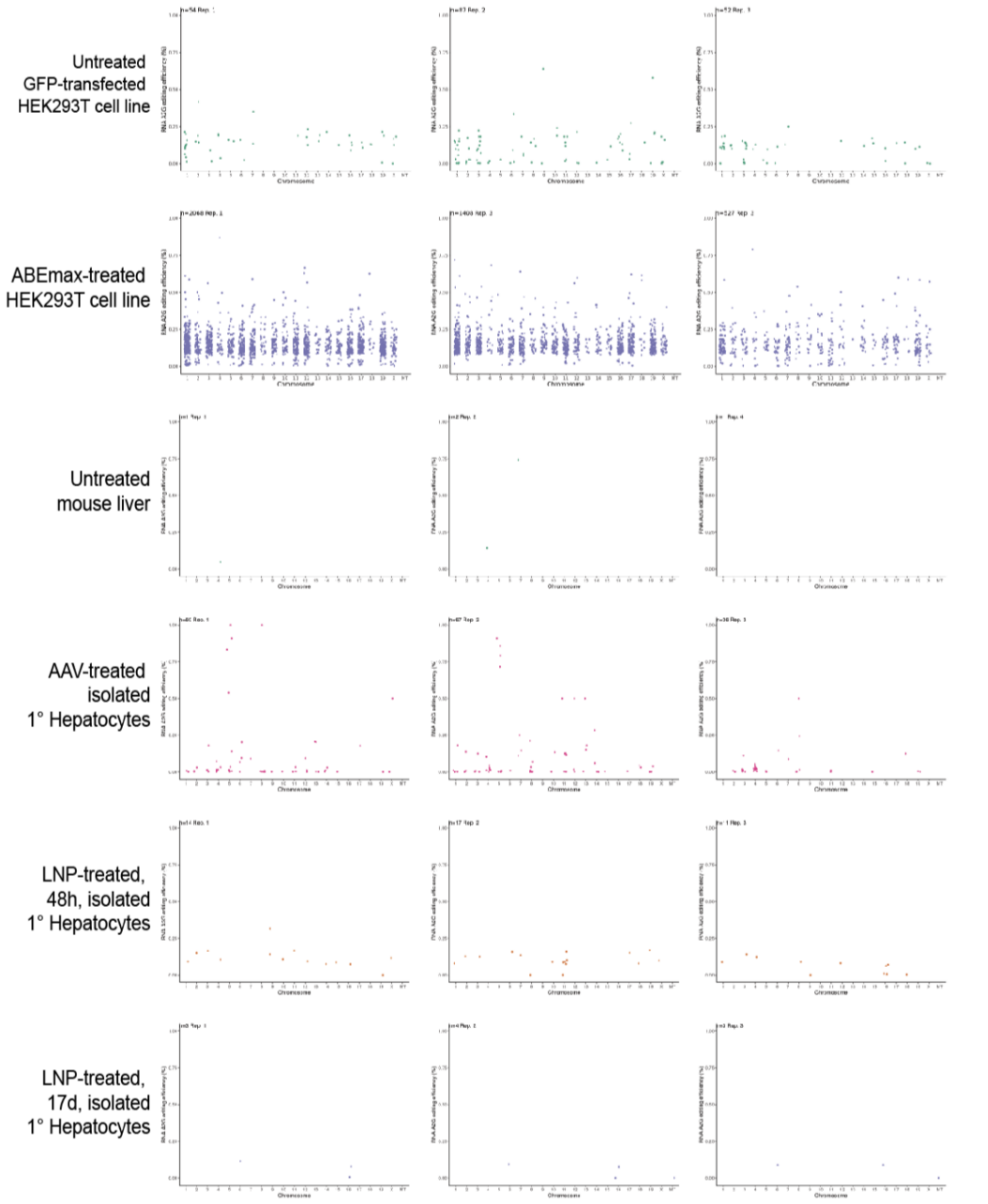

B

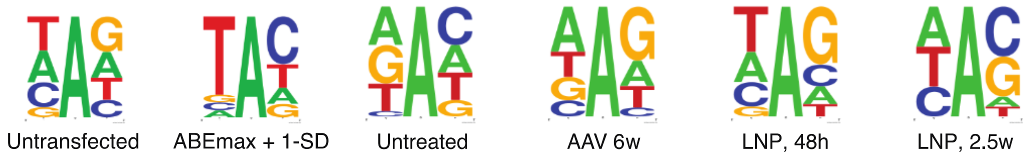

Supplementary Figure 5 | A-to-I off-target editing on the transcriptome. (A) Manhattan plots depicting A-to-I editing events on the transcriptome. Each dot represents one editing event. The chromosomal location of each edit is indicated on the x-axis. *In vitro* samples: HEK293T cells transfected with a plasmid expressing GFP (control) or plasmids expressing ABEmax and sgRNA_hP01. *In vivo* samples: Hepatocytes isolated from untreated mice (control), AAV treated mice, or LNP treated mice. (B) Trinucleotide sequence motifs for A-to-I edited adenosine of samples shown in (A). The height of each letter indicates the relative contribution of each base at this position. Ts should be read as U. As previously shown[1], the sequence preference of TadA follows a UA consensus motif.

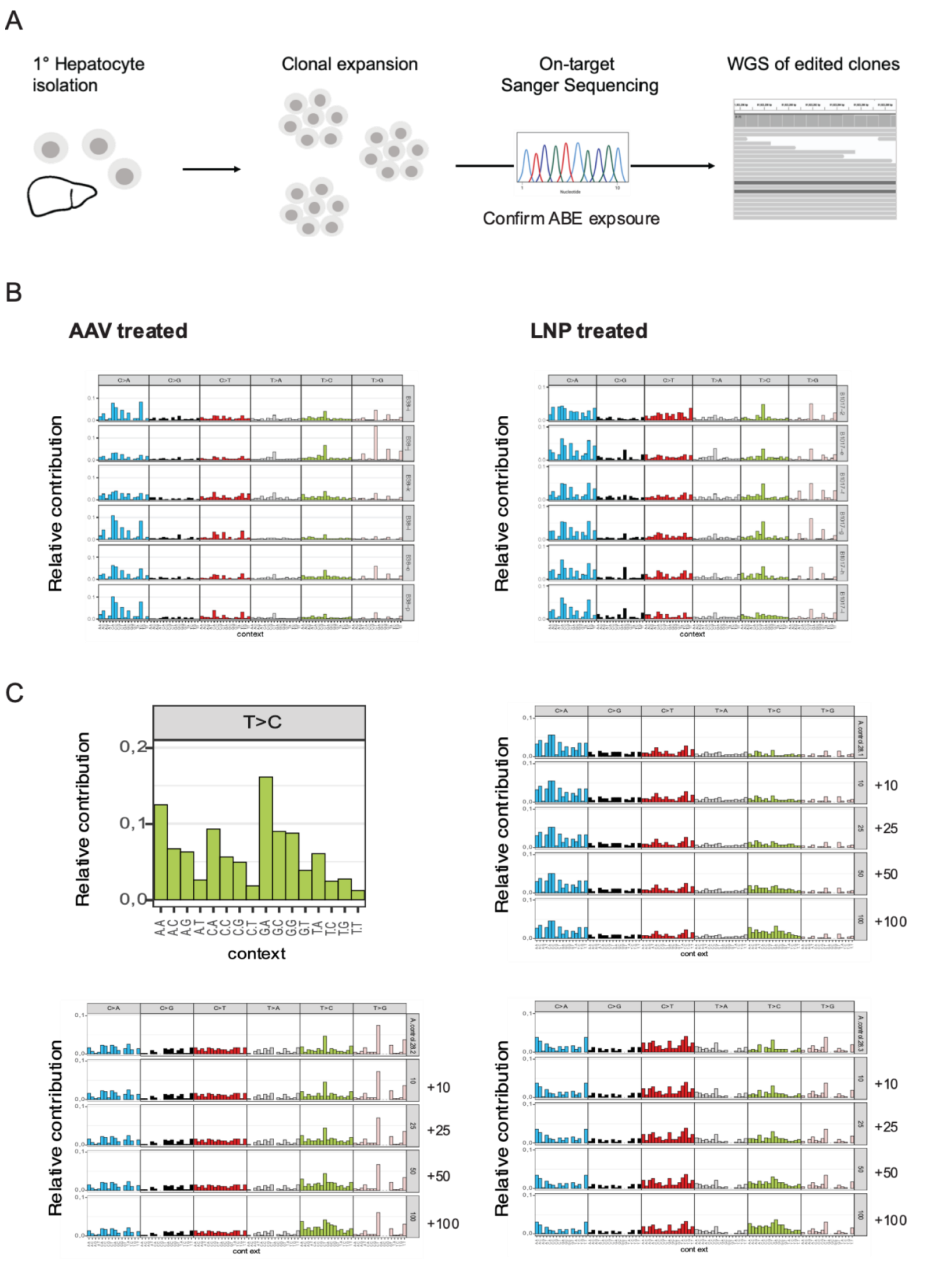

Supplementary Figure 6 | 96-nt profile plot of clonally expanded hepatocytes. (A) Schematic illustrating the procedure for assessing sgRNA-independent off-target editing events in an unbiased manner. Primary hepatocytes were isolated and clonally expanded. Only clones with on-target editing and therefore ABE exposure were selected for WGS analysis. **(**B) The frequency (y-axis) for 96 mutational types (x-axis) is shown. The x-axis shows the 5' and 3' adjacent bases to the respective motif. Each row represents one clone. Mutation types are summarized into one category: C>A includes G>T, C>G includes G>C, C>T includes G>A, T>A includes A>T, T>C includes A>G and T>G includes A>C. (C) The upper left panel shows the trinucleotide motif preference of TadA

determined by Marquart et al.[2], which was used for computational modelling of TadA signatures. Upper right and lower panels: 96-nt profile plot of control clones (upper row) with in silico added TadA signatures (lower rows). The frequency (y-axis) for 96 mutational types (x-axis) is shown. The numbers on the right indicate the number of SNVs added to the original pattern of control clones.

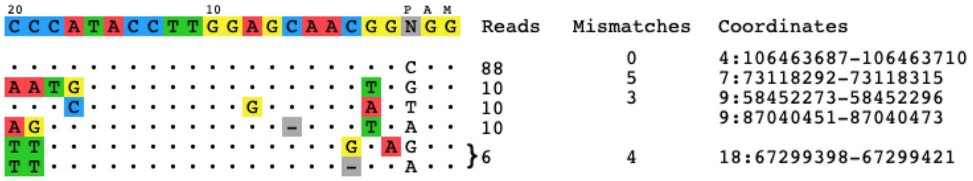

Supplementary Figure 7 | Unbiased detection of potential sgRNA_mP01 off-target sites. Off-target sites were identified using the CHANGE-seq[3] protocol. All hits were also identified by CIRCLE-seq (Fig.3).

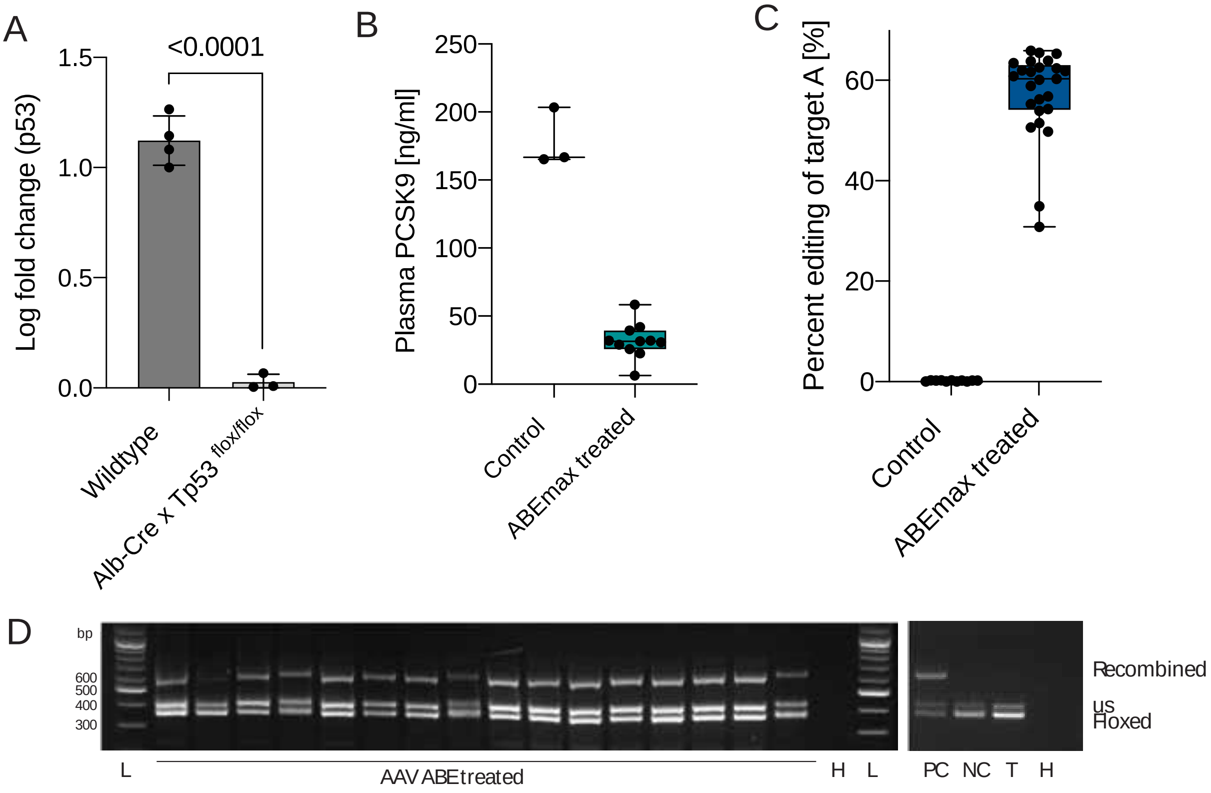

Supplementary Figure 8 | Adenine base editing in Alb-Cre x Tp53$^{flox/flox}$ mice. (A) Validation of the Tp53 knock-out in the liver. Relative p53 mRNA levels in hepatocytes isolated from Alb-Cre x Trp53$^{fl/fl}$ mice compared to Trp53$^{+/+}$ control mice as determined by RT-qPCR. Values represent mean $\pm$ s.d. of n=4, n=3 biologically independent experiments. Means were compared using a two-tailed unpaired t-test. (B) Exposure to ABEmax was confirmed by plasma reduction of PCSK9. Values represent mean $\pm$ s.d. of n=3, n=11 animals. (C) ABEmax exposure was confirmed in all treated animals by targeted amplicon sequencing. Sequencing was performed on 11 control animals and 25 treated animals. (D) Genotyping PCR validating Trp53$^{flox/flox}$ recombination in whole liver lysates of n = 16 ABE treated Alb-Cre x Trp53flox/flox mice. A band at 612 bp confirms successful recombination. Floxed Tp53 is confirmed by a band at 370 bp. An unspecific band occurs at around 400 bp. PCR validation was performed once for n=25 animals. L, ladder, H, H2O, PC, positive control (recombined mouse liver), NC, negative control (floxed mouse liver without Alb-Cre), T, tail (from Alb-Cre x Trp53$^{flox/flox}$ animal). The box plots are standard Tukey plots, in which the centerline represents the median, the lower and upper hinges represent the first and third quartiles, and the whiskers represent +/- 1.5 x the interquartile range.

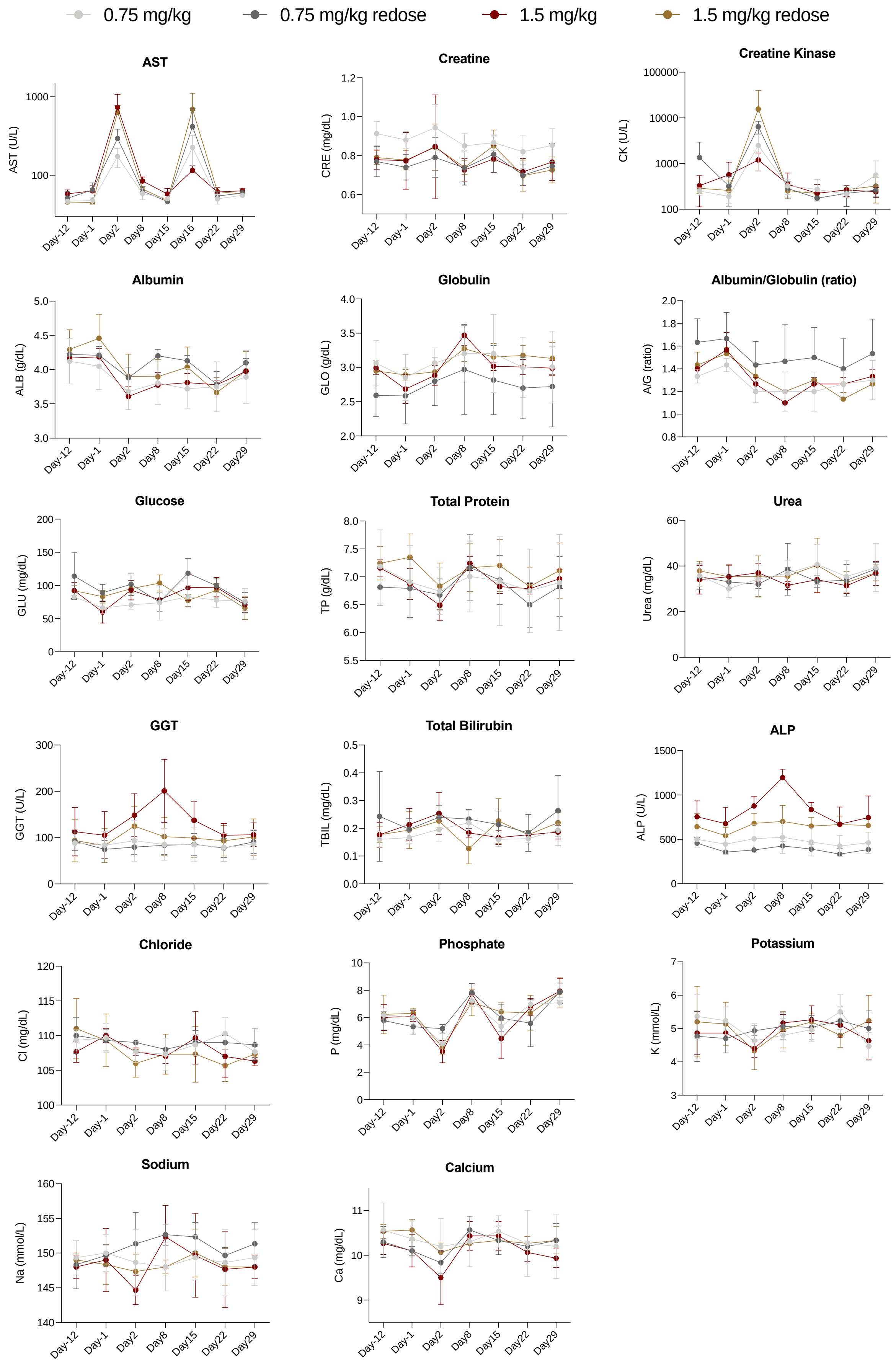

Supplementary Figure 9 | Blood samples of macaques were analyzed with a clinical chemistry panel before and during ABE treatment. Values represent mean ± s.d. of n=3 biologically independent replicates. Measurement on Day15 was taken before redosing.

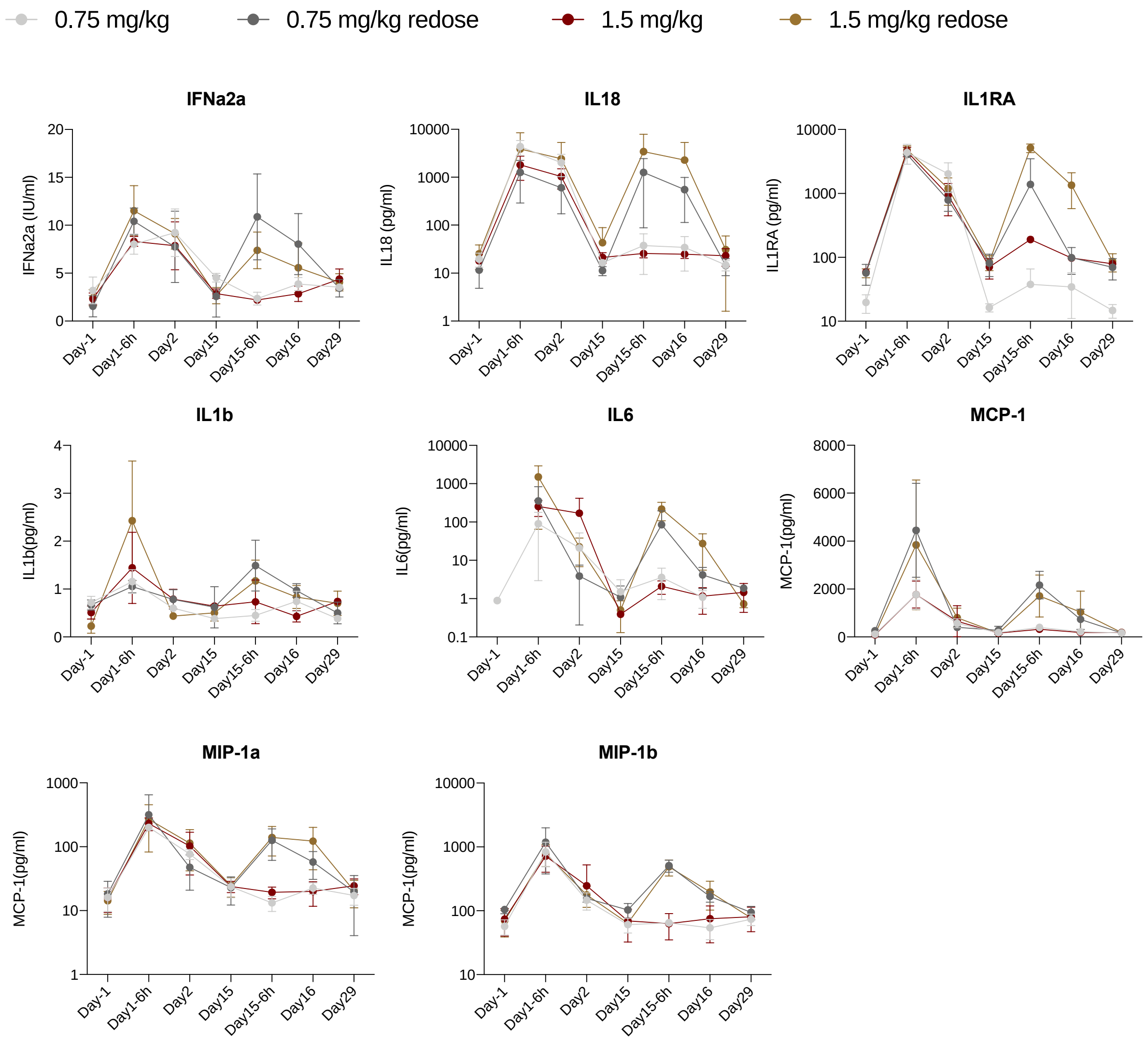

Supplementary Figure 10 | Blood samples of macaques were analyzed with an inflammatory biomarker and cytokine panel before and during ABE treatment. Values represent mean ± s.d. of n=3 biologically independent replicates. Measurement "Day15" was taken before redosing and "Day15-6h" 6 hours after redosing.

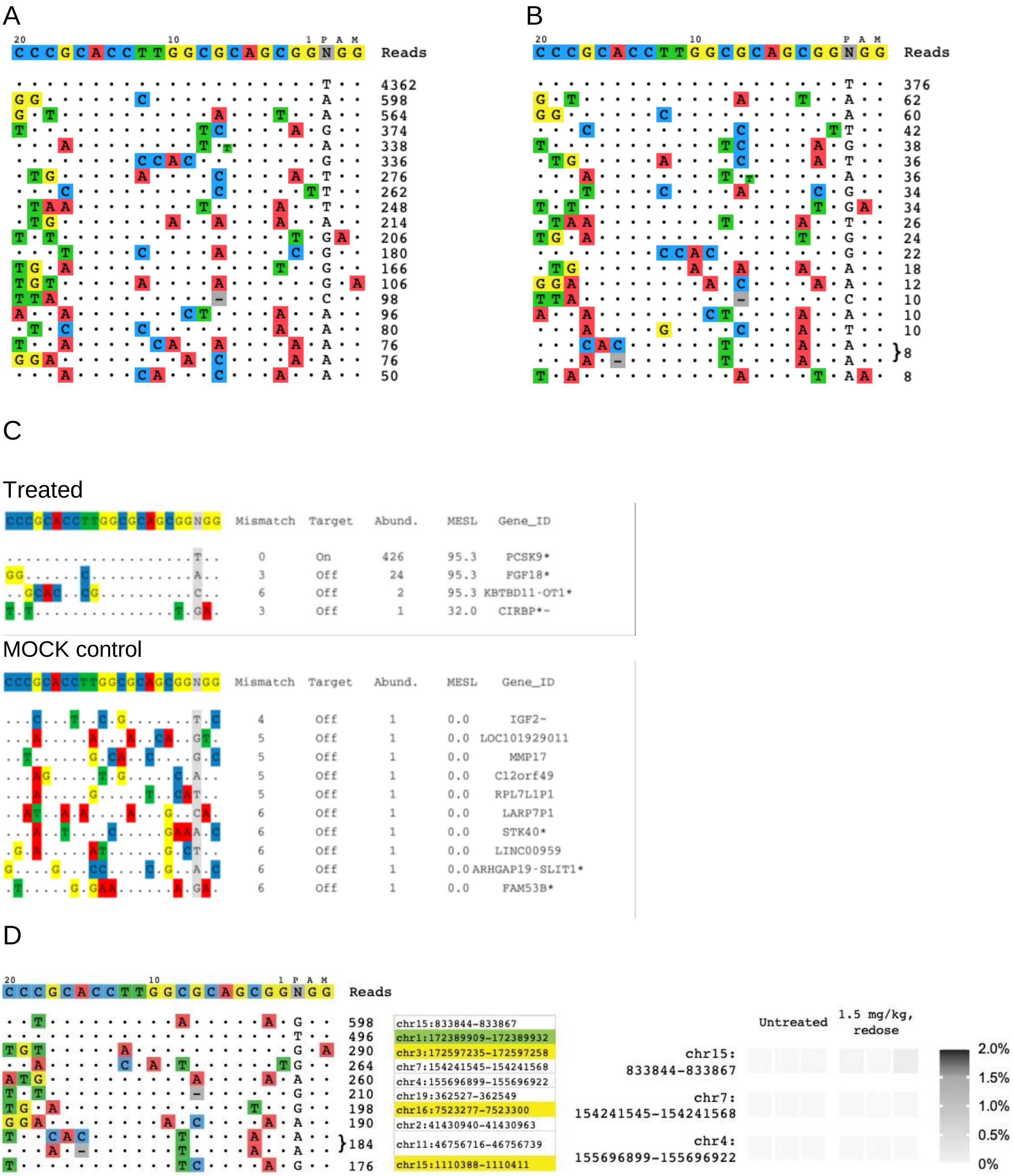

Supplementary Figure 11 | Unbiased detection of potential sgRNA_hP01 off-target sites. (A) Top hits identified by the CIRCLE-seq protocol. (B) Top hits identified by the CHANGE-seq protocol. 9 of the top 10 hits found in CIRCLE-seq were also identified by CHANGE-seq. (C) iGUIDE output from HEK293T cells transfected with sgRNA_hP01, *Sp*Cas9 and ssODN (top panel). Only sites > 2 reads were considered. The only off-target site identified by iGUIDE is also the top hit in CIRCLE-seq. In MOCK controls HEK293T cells only transfected with sgRNA. Only off-target sites where orthologous regions were found in *Macaca fascicularis* (Macaca_fascicularis_5.0/macFas5, UCSC) were selected for targeted amplicon deep sequencing. (D) Left panel: Identification of candidate off-target sites of sgRNA_hP01 in the genome of *Macaca fascicularis* by CHANGE-seq. The PCSK9 on-target site is highlighted in green. 3 of the top 10 hits were already identified in the human genome (highlighted in yellow), and analysis of these sites by targeted amplicon sequencing in treated macaques is shown in Fig. 4L. Right panel: Analysis of the top 3 off-target sites specific to the macaque genome. DNA was isolated by targeted amplicon sequencing from liver tissue of LNP-treated macaques (re-dosed with 1.5 mg/kg),

and from blood cells isolated prior to treatment. Values represent the highest A to G conversion frequency within the protospacer. n=3 biological replicates per treatment.

Supplementary Table 1 | CIRCLE-seq off-target sites for sgRNA_mP01 analyzed by targeted amplicon sequencing. Up to 6 mismatches were allowed during CIRCLE-seq analysis. The table shows the coverage of mapped reads to the respective off-target site, the matched sgRNA, and the locus of the protospacer + PAM in the mm10 reference genome.

Name	Reads	Matched sgRNA	locus
mCIRCLE1	78	CCCCTACCTTGGGGCAACAGTGG	chr9:58452273-58452296
mCIRCLE2	48	AGCATACCATGGAGCAACACGGT	chr2:17065083-17065106
mCIRCLE3	48	ACCATACCTAAGAGCAAACTGGG	chr2:25624134-25624157
mCIRCLE4	46	AGCATACCATGGAGCAACACGGT	chrX:78047768-78047791
mCIRCLE5	40	CCCATACATTGGGGCATCGGAGG	chr9:80726885-80726908
mCIRCLE6	36	ACCATACCTTGGAACAACCAAGG	chr12:16844199-16844222
mCIRCLE7	36	TTCATACCTTGGAGCAAGGAGGG	chr18:67299398-67299421
mCIRCLE8	34	AATGTACCTTGGAGCAACTGGGG	chr7:73118292-73118315
mCIRCLE9	34	GACATACCTTAAAGCAAAGGAGG	chr8:27980110-27980133
mCIRCLE10	28	CCCCTACCTTGGGGCAACAG	chr9:87040451-87040473

Supplementary Table 2 | CIRCLE-seq off-target sites for sgRNA_hP01 analyzed by targeted amplicon sequencing. Loci in the human genome and orthologous sites in the genome of *Macaca fascicularis* are indicated. Up to 6 mismatches were allowed for CIRCLE-seq analysis. The table shows the coverage of mapped reads to the respective off-target site, the matched sgRNA, and the locus of the protospacer + PAM in the mm10 reference genome. Mismatches between human and macaque sequences are highlighted in red. Only sites with less or equal 3 mismatches were selected of deep-sequencing.

No	Reads	sequence human	sequence ce-macaca	MM	locus human	locus ce-macaca
1	598	GGCGCACCCTGGCGCAGCGGAGG	GGCGCGCCCTGGCGCAGCGGAGG	2	chr5:171451187-171451210	chrUn_EK146647:1899-1921
2	564	GCTGCACCTTGGCACAGTGGAGG	GCTACACCTTGGCACAGTGGCAG	3	chr19:50490617-50490640	chr19:51326546-51326568
3	374	TCCGCACCTTGGTCCAGCAGGGG	TCCGCACCTTGGTCCAGCAGGGG	0	chr9:137167998-137168021	chr15:1110389-1110411
4	338	CCCACACCTTGGTGTCAGCGGAGG	CCCACAACTTGGTGTCAGTGGAGG	2	chr3:14242672-14242696	chr11:86241801 -86241824
5	276	CTGGCACCATGGCCCAGCAGTGG	CTGGCACCATGGCCCAGCAGTGG	0	chr1:25103341-25103364	chr1:203253892-203253914
6	262	CCCCCACCTTGGCCCAGCGTTGG	CCCCTACCTTGGCCCAGCGTTGG	1	chr19:6431798-6431821	chr19:6608322-6608344
7	166	TGCACACCTTGGCGCAGTGGGGG	TGCACACCTTGGCGCAGTGGGGG	0	chr17:7462658-7462681	chr16:7523278-7523300
8	106	TGTGCACCATGGCACAGCGGGGA	TGTGCACCATGGCGCAGCGGGGA	1	chr7:139596894-139596917	chr3:172597236-172597258

Supplementary Table 3 | Additional mouse sgRNAs tested in this study. Spliceosome-recognition sites with target adenine are underlined. PAM site in blue.

mPCSK9-4SA spacer sequence + PAM	ggaagatggaagcagccaggtgg	Spliceosome-recognition site, underlined
mPCSK9-6SA spacer sequence + PAM	ttgcaggcctggagtttattcgg	Spliceosome-recognition site, underlined
mPCSK9-6SD spacer sequence + PAM	cctacctctggagcagaagctgg	Spliceosome-recognition site, underlined

mPCSK9-7SA spacer sequence + PAM	tgccaggtcatcacagtcgggg	Spliceosome-recognition site, underlined
mPCSK9-8SD spacer sequence + PAM	ctcacctgtctcatgggtgctgg	Spliceosome-recognition site, underlined
mPCSK9-11SA spacer sequence + PAM	tctaggctgcagcttccattggg	Spliceosome-recognition site, underlined

Supplementary Table 4 | On-target sequencing of DNA from liver biopsies of treated macaques. The average editing efficiency per animal is summarized in the very right column.

Animal ID	Caudate	Left	Quadrate_1	Quadrate_2	Right_1	Right_2	Average
101	1.29670943	1.15042058	1.39945239	1.26750282	1.32621705	1.13012593	1.26173803
102	2.58773485	1.87332739	1.75054705	1.76981542	2.27347611	2.90997706	2.19414631
103	2.64955252	3.3873816	2.82352941	4.29378531	1.91238604	2.83550546	2.98369006
201	2.73238682	2.3881661	2.22363405	2.85744978	1.50018752	2.20918622	2.31850175
202	6.68384142	3.97887324	6.35316699	7.19582851	3.56245124	4.65335639	5.4045863
203	2.88557214	2.25580538	1.99554393	3.56302916	2.35669758	1.95937628	2.50267075
301	13.3949928	33.7160296	27.2890485	18.3094262	21.9637273	16.3546374	21.837977
302	37.1537853	29.3938188	22.790906	31.9125174	22.9177391	22.8024607	27.8285379
303	31.0052382	44.3266616	32.0111732	28.1796311	39.2398815	26.5566506	33.553206
401	19.4481748	19.0970275	44.3494197	18.5862069	22.0233902	20.847502	24.0586202
402	29.3512122	25.4981425	27.4274662	19.4710779	16.4604507	18.9490135	22.8595605
404	20.2874207	18.2348651	16.2294732	31.3399554	34.8323516	34.3689969	25.8821771

Supplementary Table 5 | Sanger and RT-qPCR primers used in this study. PCR amplification was performed using respective forward (fw) and reverse (rev) primers. Sanger sequencing was performed using the respective in-sequence primer (in_seq). HKG, housekeeping gene

hPCSK9_P01_Sanger Fw	ACTTCAGCTCCTGCACAGTC	Sanger, amplicon primer, human
hPCSK9_P01_Sanger Rev	ACCTTCCCACTGAATAGCGC	Sanger, amplicon primer, human
hPCSK9_P01_Sanger in_seq	ACCTGCACTCCACTTCCTCTC	Sanger, in-sequence primer, human
NHP_PCSK9_Sanger_FW	ACTCCAGCTCCTGCACAGTC	Sanger, amplicon primer, macaque
NHP_PCSK9_Sanger_Rev	GCCTTCCCACTGAATAGCGC	Sanger, amplicon primer, macaque
NHP_PCSK9_Sanger_in_seq	CTGATGGGTACCGTCAGCTC	Sanger, in-sequence primer, macaque
mPCSK9_P01_Sanger Fw	CTTGGCTCCCCAGAGACATC	Sanger, amplicon primer, mouse
mPCSK9_P01_Sanger Rev	CTAAGTCTTGCCCTCGCCTC	Sanger, amplicon primer, mouse
mPCSK9_P01_Sanger in_seq	ACCCACTGCTCTGCGTGGCT	Sanger, in-sequence primer, mouse

PCSK9_4SA_Sanger Fw	CATATGTTTGGGAGGTTGGCT	Sanger, amplicon primer
PCSK9_4SA_Sanger Rev	GTACCTCTGCCCACCTTCAC	Sanger, amplicon primer
PCSK9_6SA/6SD_Sanger Fw	AGGTTAAGCATCCGAGCACC	Sanger, amplicon primer
PCSK9_6SA/6SD_Sanger Rev	AATGGCTCAGGGGATTGTGG	Sanger, amplicon primer
PCSK9_7SA_Sanger Fw	TACCCTAGAACCTGGGCTCC	Sanger, amplicon primer
PCSK9_7SA_Sanger Rev	TGTGAGGTCCCACTCTGTGA	Sanger, amplicon primer
PCSK9_8SD_Sanger Fw	TGGGTCTACTAGGGAAGGAT	Sanger, amplicon primer
PCSK9_8SD_Sanger Rev	CACCCGCCAGAGATGTTAGG	Sanger, amplicon primer
PCSK9_11SA_Sanger Fw	GGGAAGGGACTCAAAGAGGC	Sanger, amplicon primer
PCSK9_11SA_Sanger Rev	ACCTGAGATCCCATGCTCCT	Sanger, amplicon primer
PCSK9_4SA_Sanger in_seq	GGGGAGATTTCCCATGAGCC	Sanger, in-sequence primer
PCSK9_6SA/6SD_Sanger in_seq	TGTGTCTCTGAGGGGAGGAG	Sanger, in-sequence primer
PCSK9_7SA_Sanger in_seq	TGGAGGAGGTGAGATGCAGA	Sanger, in-sequence primer
PCSK9_8SD_Sanger in_seq	CATTGTGGCTCGGATGCTGA	Sanger, in-sequence primer
PCSK9_11SA_Sanger in_seq	AAGAGGTTGCATGGCTCTCC	Sanger, in-sequence primer

Trp53 Genotyping Primer A	CAC AAA AAC AGG TTA AAC CCA G	Assessment of recombined, wildtype or floxed Trp53
Trp53 Genotyping Primer B	AGC ACA TAG GAG GCA GAG AC	Assessment of recombined, wildtype or floxed Trp53
Trp53 Genotyping Primer D	GAA GAC AGA AAA GGG GAG GG	Assessment of recombined, wildtype or floxed Trp53
Cre 351 Fw	CGA CCA GGT TCG TTC ACT CA	Assessment of presence of Albumin-Cre
Cre 351 Rev	CGA GTT GAT AGC TGG CTG GT	Assessment of presence of Albumin-Cre

mPCKS9_RT-qPCR Fw	TATGCCGTCGCGAGATGCTG	RT-qPCR Primer mouse PCSK9
mPCKS9_RT-qPCR Rev	TGACGTCTGGACCTCAGC	RT-qPCR Primer mouse PCSK9
mB2M FW	TTCTGGTGCTTGTCTCACTGA	RT-qPCR Primer mouse HKG
mB2M Rev	CAGTATGTTCGGCTTCCCATTC	RT-qPCR Primer mouse HKG
hPCSK9_RT-qPCR Fw	ATCCACGCTTCCTGCTGC	RT-qPCR Primer human PCSK9
hPCSK9_RT-qPCR Rev	CACGGTCACCTGCTCCTG	RT-qPCR Primer human PCSK9
hGAPDH Fw	GTCCACTGGCGTGTTCACCA	RT-qPCR Primer human HKG
hGAPDH Rev	GTGGCAGTGATGGCATGGAC	RT-qPCR Primer human HKG
P53 Exon 4/5-spanning Fw	CTTCCTGCAGTCTGGGACAG	RT-qPCR Primer mouse p53
Trp53 Exon 4/5-spanning Rev	TCTCACGACCTCCGTCATGT	RT-qPCR Primer mouse p53

Supplementary Table 6 | High throughput (HTS) sequencing primers used in this study

mPCSK9_HTS_Fw	CTTTCCCTACACGACGCTCTTCCGATCTNNNNNNCTCCCGTCCCAGGAGGAT	HTS for on-target mus musculus gDNA
mPCSK9_HTS_Rev	GGAGTTCAGACGTGTGCTCTTCCGATCTNNNNNNCGGCTAGATGAGCAGAGAAGA	HTS for on-target mus musculus gDNA
NHP_PCSK9_HTS_Fw	CTTTCCCTACACGACGCTCTTCCGATCTACCTGCACCCCACTTCCTCTC	HTS for on-target macaca fascicularis gDNA
NHP_PCSK9_HTS_Rev	GGAGTTCAGACGTGTGCTCTTCCGATCTCGTTCCGAGGAGGACGGC	HTS for on-target macaca fascicularis gDNA
hPCSK9_HTS_Fw	CTTTCCCTACACGACGCTCTTCCGATCTNNNNNNAACCACAGCCACCTTCCAC	HTS for on-target homo sapiens gDNA
hPCSK9_HTS_Rev	GGAGTTCAGACGTGTGCTCTTCCGATCTNNNNNNAAACTGAGGCCCGAGAGG	HTS for on-target homo sapiens gDNA

mCIRCLE1 Fw	CTTTCCCTACACGACGCTCTTCCGATCTATGTCACTTCTGCACACCAAGA	HTS for off-target site chr9:58452273-58452296
mCIRCLE1 Rev	GGAGTTCAGACGTGTGCTCTTCCGATCTCCTTCCCAGGAGAATCTGTCAC	HTS for off-target site chr9:58452273-58452296
mCIRCLE2 Fw	CTTTCCCTACACGACGCTCTTCCGATCTAGTGCCTAGGCGTAAATCTC	HTS for off-target site chr2:17065083-17065106
mCIRCLE2 Rev	GGAGTTCAGACGTGTGCTCTTCCGATCTCAGAGAGACCTCAAGATAGC	HTS for off-target site chr2:17065083-17065106
mCIRCLE3 Fw	CTTTCCCTACACGACGCTCTTCCGATCTCCAAGGATGGCTCTCAGCAA	HTS for off-target site chr2:25624134-25624157
mCIRCLE3 Rev	GGAGTTCAGACGTGTGCTCTTCCGATCTGATGCTCACACTGGGCAGAA	HTS for off-target site chr2:25624134-25624157
mCIRCLE4 Fw	CTTTCCCTACACGACGCTCTTCCGATCTTCCTAACTTGTTCCACGAGGC	HTS for off-target site chrX:78047768-78047791
mCIRCLE4 Rev	GGAGTTCAGACGTGTGCTCTTCCGATCTGGCACAATGGGGTCCTAACA	HTS for off-target site chrX:78047768-78047791
mCIRCLE5 Fw	CTTTCCCTACACGACGCTCTTCCGATCTAGAGATGGTTGGGGAAGCAC	HTS for off-target site chr9:80726885-80726908
mCIRCLE5 Rev	GGAGTTCAGACGTGTGCTCTTCCGATCTCCATACATTGGGGCATCGGA	HTS for off-target site chr9:80726885-80726908
mCIRCLE6 Fw	CTTTCCCTACACGACGCTCTTCCGATCTTCTTACAGGAGGCTCTGGCT	HTS for off-target site chr12:16844199-16844222
mCIRCLE6 Rev	GGAGTTCAGACGTGTGCTCTTCCGATCTTTCCAGGACAGTCAGGGCTA	HTS for off-target site chr12:16844199-16844222
mCIRCLE7 Fw	CTTTCCCTACACGACGCTCTTCCGATCTAGGTGACTTGTCTTCCTTGTGT	HTS for off-target site chr18:67299398-67299421
mCIRCLE7 Rev	GGAGTTCAGACGTGTGCTCTTCCGATCTGACATCTGGCCACTGGTGTG	HTS for off-target site chr18:67299398-67299421
mCIRCLE8 Fw	CTTTCCCTACACGACGCTCTTCCGATCTATGAGACACTCTAGCCCTTCAG	HTS for off-target site chr7:73118292-73118315
mCIRCLE8 Rev	GGAGTTCAGACGTGTGCTCTTCCGATCTATACCTACACTTCTCAAGCCCAC	HTS for off-target site chr7:73118292-73118315
mCIRCLE9 Fw	CTTTCCCTACACGACGCTCTTCCGATCTAGTTGTTTGTTCAACTTAAAGGACA	HTS for off-target site chr8:27980110-27980133
mCIRCLE9 Rev	GGAGTTCAGACGTGTGCTCTTCCGATCTTCCCTTGCCACTGTCATTATCTT	HTS for off-target site chr8:27980110-27980133
mCIRCLE10 Fw	CTTTCCCTACACGACGCTCTTCCGATCTGCTTCCCAAGAGCCAAATGTC	HTS for off-target site chr9:87040451-87040473
mCIRCLE10 Rev	GGAGTTCAGACGTGTGCTCTTCCGATCTATAACGTTTGTGGGGTTGTGC	HTS for off-target site chr9:87040451-87040473

hCIRCLE1 Fw	CTTTCCCTACACGACGCTCTTCCGATCTCGCCCTCTCTCCCGTCATTA	HTS for off-target site chr5:171451187-171451210/chrUn_EK146647:1899-1921
hCIRCLE1 Rev	GGAGTTCAGACGTGTGCTCTTCCGATCTGACAGGAGGCTGGCAAGG	HTS for off-target site chr5:171451187-171451210/chrUn_EK146647:1899-1921
hCIRCLE2 Fw	CTTTCCCTACACGACGCTCTTCCGATCTTCACGTCCTTTCTCCCAGG	HTS for off-target site chr19:50490617-50490640/chr19:51326546-51326568
hCIRCLE2 Rev	GGAGTTCAGACGTGTGCTCTTCCGATCTAAGGATGTCAGGGACTAGGC	HTS for off-target site chr19:50490617-50490640/chr19:51326546-51326568
hCIRCLE3 Fw	CTTTCCCTACACGACGCTCTTCCGATCTGACTCAGCTCTGCCCCGTC	HTS for off-target site chr9:137167998-137168021/chr15:1110389-1110411
hCIRCLE3 Rev	GGAGTTCAGACGTGTGCTCTTCCGATCTGATGGGTCCTGCTGCTCG	HTS for off-target site chr9:137167998-137168021/chr15:1110389-1110411
hCIRCLE4 Fw	CTTTCCCTACACGACGCTCTTCCGATCTTAGAAGTCCAGGTTTCCCAC	HTS for off-target site chr3:14242672-14242696/chr11:86241801 -86241824
hCIRCLE4 Rev	GGAGTTCAGACGTGTGCTCTTCCGATCTAAACTGTCAGTGAGACTGCT	HTS for off-target site chr3:14242672-14242696/chr11:86241801 -86241824
hCIRCLE5 Fw	CTTTCCCTACACGACGCTCTTCCGATCTGGTTCTCAAGCAAGGCTGTC	HTS for off-target site chr1:25103341-25103364/chr1:203253892-203253914
hCIRCLE5 Rev	GGAGTTCAGACGTGTGCTCTTCCGATCTCTGCTCGCTGGAAGTTGGAG	HTS for off-target site chr1:25103341-25103364/chr1:203253892-203253914
hCIRCLE6 Fw	CTTTCCCTACACGACGCTCTTCCGATCTGCATTAGAGGCATGAGCCAC	HTS for off-target site chr19:6431798-6431821/chr19:6608322-6608344
hCIRCLE6 Rev	GGAGTTCAGACGTGTGCTCTTCCGATCTTGGAGTTGGGCTCTCCTGGA	HTS for off-target site chr19:6431798-6431821/chr19:6608322-6608344
hCIRCLE7 Fw	CTTTCCCTACACGACGCTCTTCCGATCTGCTGCCCTTGGAATAGGCAA	HTS for off-target site chr17:7462658-7462681/chr16:7523278-7523300
hCIRCLE7 Rev	GGAGTTCAGACGTGTGCTCTTCCGATCTCCTCCACCCGCTTTACCTG	HTS for off-target site chr17:7462658-7462681/chr16:7523278-7523300
hCIRCLE8 Fw	CTTTCCCTACACGACGCTCTTCCGATCTGGAATGACAATTGTCTGCCG	HTS for off-target site chr7:139596894-139596917/chr3:172597236-172597258
hCIRCLE8 Rev	GGAGTTCAGACGTGTGCTCTTCCGATCTGAGGTGTCCTCCTCTCAGGC	HTS for off-target site chr7:139596894-139596917/chr3:172597236-172597258

NHP_CHANGE_1 Fw	CTTTCCCTACACGACGCTCTTCCGATCTGATGCAGATGGGGTGACCAA	HTS for off-target site chr15:833844-833867
NHP_CHANGE_1 Rev	GGAGTTCAGACGTGTGCTCTTCCGATCTCCCAGTTTCGAGTGCCAGG	HTS for off-target site chr15:833844-833867
NHP_CHANGE_2 Fw	CTTTCCCTACACGACGCTCTTCCGATCTCCTCCAGCAACAGCTCTCTAAA	HTS for off-target site chr7:154241545-154241568
NHP_CHANGE_2 Rev	GGAGTTCAGACGTGTGCTCTTCCGATCTTCATTGTAGCAGAGGACGGTGT	HTS for off-target site chr7:154241545-154241568
NHP_CHANGE_3 Fw	CTTTCCCTACACGACGCTCTTCCGATCTCCAGGGAGAGCCTGGGT	HTS for off-target site c hr4:155696899-155696922
NHP_CHANGE_3 Rev	GGAGTTCAGACGTGTGCTCTTCCGATCTCGTTGGACGGGGCGG	HTS for off-target site chr4:155696899-155696922

Supplementary Table 7 | smFISH probe sequences against ABEmax mRNA

tctttggtgactcgaactcg	agctggatgaacagcttgtc	tcactttgtcgtcgaacagg	tacacgaactcgctttccag
catccaatactcgtggctaa	agaaacaggtcggcgtactg	catgaagtttctgttggcga	ctcggtctttttcacgatat
cgatcactctattgttgtgc	tcgtcgtatctcttgatcat	agattggcaatgtgctcgtg	caggatagactctttgctga
atcgatcaggcggtaattct	tctttgtacttctcaggcag	ccatttcgatcacgatgttc	tttctggcgatcagcttatc
cataatttcggcatgggctg	ttcttgctctggtcgaagaa	cgatatggtccacatcgtag	cactcttcagtttcttggac
caaggctcgaatgtcacgta	tgggtaaaaatcttcctgcc	atggagtcgtccttcagaaa	tcgatgggattcttctcgaa
cgatcctagagtggatcatg	cttatcgaagttggtcatcc	gtcgaactttctctgggtaa	acttaggcagcttgatgatc
aatttcgacgcggtgattca	cacggtgaagtactcgtaca	cgtcgtacttagtgttcatc	agttcacatatttggagggc
attgaacacctgtctaggca	tatttcactttggtcagctc	ttcagggtgatcactttcac	tgatctgctcgatgatctcg
caccttgaatttcttgctgg	tcagcagatcgtggtatgtg	aaatccttccggaaatcgga	ggatcactctcttggagaac
gctgaagatctcttgcagat	gaagtccttgtccttgataa	gatctcgcgcactttgtaaa	ccagattggtcagggtaaac
agtctgtggaagaagctgtc	atatcttccagaatgtcctc	agggtactttttgatcaggg	caaagtacttgaaggcggca

Supplementary Note 1 | Amino Acid sequences of AAV vectors used in this study

N-int-ABEmax/-dCas: NLS - TadA-TadA* - linker - N-term-nCas9 - DnaE Npu N-intein

MKRTADGSEFESPKKKRKVSEVEFSHEYWMRHALTLAKRAWDEREVPVGAVLVHNNRVIGEGWNRPIGRHDPTAHAEIMALRQGGLV
MQNYRLIDATLYVTLEPCVMCAGAMIHSRIGRVVFGARDAKTGAAGSLMDVLHHPGMNHRVEITEGILADECAALLSDFFRMRRQEIKA
QKKAQSSTDSGGSSGGSSGSETPGTSESATPESSGGSSGGSSEVEFSHEYWMRHALTLAKRARDEREVPVGAVLVLNNRVIGEGWNRAIG
LHDPTAHAEIMALRQGGLVMQNYRLIDATLYVTFEPCVMCAGAMIHSRIGRVVFGVRNAKTGAAGSLMDVLHYPGMNHRVEITEGILA
DECAALLCYFFRMPRQVFNAQKKAQSSTDSGGSSGGSSGSETPGTSESATPESSGGSSGGSDKKYSIGLDIGTNSVGWAVITDEYKVPSKKF
KVLGNTDRHSIKKNLIGALLFDSGETAEATRLKRTARRRYTRRKNRICYLQEIFSNEMAKVDDSFFHRLEESFLVEEDKKHERHPIFGNIVDEV
AYHEKYPTIYHLRKKLVDSTDKADLRLIYLALAHMIKFRGHFLIEGDLNPDNSDVDKLFIQLVQTYNQLFEENPINASGVDAKAILSARLSKSR
RLENLIAQLPGEKKNGLFGNLIALSLGLTPNFKSNFDLAEDAKLQLSKDTYDDDLDNLLAQIGDQYADLFLAAKNLSDAILLSDILRVNTEITKA
PLSASMIKRYDEHHQDLTLLKALVRQQLPEKYKEIFFDQSKNGYAGYIDGGASQEEFYKFIKPILEKMDGTEELLVKLNREDLLRKQRTFDNG
SIPHQIHLGELHAILRRQEDFYPFLKDNREKIEKILTFRIPYYVGPLARGNSRFAWMTRKSEETITPWNFEEVVDKGASAQSFIERMTNFDKN
LPNEKVLPKHSLLYEYFTVYNELTKVKYVTEGMRKPAFLSGEQKKAIVDLLFKTNRKVTVKQLKEDYFKKIECLSYETEILTVEYGLLPIGKIVEK
RIECTVYSVDNNGNIYTQPVAQWHDRGEQEVFEYCLEDGSLIRATKDHKFMTVDGQMLPIDEIFERELDLMRVDNLPN*

N-int-ABE8e/-dCas: NLS – TadA8e - linker - N-term-nCas9 - DnaE Npu N-intein

MKRTADGSEFESPKKKRKVSEVEFSHEYWMRHALTLAKRARDEREVPVGAVLVLNNRVIGEGWNRAIGLHDPTAHAEIMALRQGGLVM
QNYRLIDATLYVTFEPCVMCAGAMIHSRIGRVVFGVRNSKRGAAGSLMNVLNYPGMNHRVEITEGILADECAALLCDFYRMPRQVFNAQ
KKAQSSINSGGSSGGSSGSETPGTSESATPESSGGSSGGSSEVEFSHEYWMRHALTLAKRARDEREVPVGAVLVLNNRVIGEGWNRAIGL
HDPTAHAEIMALRQGGLVMQNYRLIDATLYVTFEPCVMCAGAMIHSRIGRVVFGVRNAKTGAAGSLMDVLHYPGMNHRVEITEGILAD
ECAALLCYFFRMPRQVFNAQKKAQSSTDSGGSSGGSSGSETPGTSESATPESSGGSSGGSDKKYSIGLDIGTNSVGWAVITDEYKVPSKKFK
VLGNTDRHSIKKNLIGALLFDSGETAEATRLKRTARRRYTRRKNRICYLQEIFSNEMAKVDDSFFHRLEESFLVEEDKKHERHPIFGNIVDEVA
YHEKYPTIYHLRKKLVDSTDKADLRLIYLALAHMIKFRGHFLIEGDLNPDNSDVDKLFIQLVQTYNQLFEENPINASGVDAKAILSARLSKSRRL
ENLIAQLPGEKKNGLFGNLIALSLGLTPNFKSNFDLAEDAKLQLSKDTYDDDLDNLLAQIGDQYADLFLAAKNLSDAILLSDILRVNTEITKAPL
SASMIKRYDEHHQDLTLLKALVRQQLPEKYKEIFFDQSKNGYAGYIDGGASQEEFYKFIKPILEKMDGTEELLVKLNREDLLRKQRTFDNGSI
PHQIHLGELHAILRRQEDFYPFLKDNREKIEKILTFRIPYYVGPLARGNSRFAWMTRKSEETITPWNFEEVVDKGASAQSFIERMTNFDKNLP
NEKVLPKHSLLYEYFTVYNELTKVKYVTEGMRKPAFLSGEQKKAIVDLLFKTNRKVTVKQLKEDYFKKIECLSYETEILTVEYGLLPIGKIVEKRIE
CTVYSVDNNGNIYTQPVAQWHDRGEQEVFEYCLEDGSLIRATKDHKFMTVDGQMLPIDEIFERELDLMRVDNLPN*

C-int-ABEmax/ABE8e: DnaE Npu C-intein -C-term-nCas9, NLS-(GSG-P2A)-tagRFP

MIKIATRKYLGKQNVYDIGVERDHNFALKNGFIASCFDSVEISGVEDRFNASLGTYHDLLKIIKDKDFLDNEENEDILEDIVLTLTLFEDREMIE
ERLKTYAHLFDDKVMKQLKRRRYTGWGRLSRKLINGIRDKQSGKTILDFLKSDGFANRNFMQLIHDDSLTFKEDIQKAQVSGQGDSLHEHI
ANLAGSPAIKKGILQTVKVVDELVKVMGRHKPENIVIEMARENQTTQKGQKNSRERMKRIEEGIKELGSQILKEHPVENTQLQNEKLYLYYL
QNGRDMYVDQELDINRLSDYDVDHIVPQSFLKDDSIDNKVLTRSDKNRGKSDNVPSEEVVKKMKNYWRQLLNAKLITQRKFDNLTKAER
GGLSELDKAGFIKRQLVETRQITKHVAQILDSRMNTKYDENDKLIREVKVITLKSKLVSDFRKDFQFYKVREINNYHHAHDAYLNAVVGTALI
KKYPKLESEFVYGDYKVYDVRKMIAKSEQEIGKATAKYFFYSNIMNFFKTEITLANGEIRKRPLIETNGETGEIVWDKGRDFATVRKVLSMPQ
VNIVKKTEVQTGGFSKESILPKRNSDKLIARKKDWDPKKYGGFDSPTVAYSVLVVAKVEKGKSKKLKSVKELLGITIMERSSFEKNPIDFLEAK
GYKEVKKDLIIKLPKYSLFELENGRKRMLASAGELQKGNELALPSKYVNFLYLASHYEKLKGSPEDNEQKQLFVEQHKHYLDEIIEQISEFSKRV
ILADANLDKVLSAYNKHRDKPIREQAENIIHLFTLTNLGAPAAFKYFDTTIDRKRYTSTKEVLDATLIHQSITGLYETRIDLSQLGGDSGGSKRT
ADGSEFEPKKKRKVGSGATNFSLLKQAGDVEENPGPMVSKGEELIKENMHMKLYMEGTVNNHHFKCTSEGEGKPYEGTQTMRIKVVEG
GPLPFAFDILATSFMYGSRTFINHTQGIPDFFKQSFPEGFTWERVTTYEDGGVLTATQDTSLQDGCLIYNVKIRGVNFPSNGPVMQKKTLG
WEANTEMLYPADGGLEGRSDMALKLVGGGHLICNFKTTYRSKKPAKNLKMPGVYYVDHRLERIKEADKETYVEQHEVAVARYCDLPSKL
GHKLN*

C-int-ABEmax/ABE8e-dCas: DnaE Npu C-intein -C-term-nCas9, NLS-(GSG-P2A)-tagRFP

MIKIATRKYLGKQNVYDIGVERDHNFALKNGFIASCFDSVEISGVEDRFNASLGTYHDLLKIIKDKDFLDNEENEDILEDIVLTLTLFEDREMIE
ERLKTYAHLFDDKVMKQLKRRRYTGWGRLSRKLINGIRDKQSGKTILDFLKSDGFANRNFMQLIHDDSLTFKEDIQKAQVSGQGDSLHEHI
ANLAGSPAIKKGILQTVKVVDELVKVMGRHKPENIVIEMARENQTTQKGQKNSRERMKRIEEGIKELGSQILKEHPVENTQLQNEKLYLYYL
QNGRDMYVDQELDINRLSDYDVDAIVPQSFLKDDSIDNKVLTRSDKNRGKSDNVPSEEVVKKMKNYWRQLLNAKLITQRKFDNLTKAER
GGLSELDKAGFIKRQLVETRQITKHVAQILDSRMNTKYDENDKLIREVKVITLKSKLVSDFRKDFQFYKVREINNYHHAHDAYLNAVVGTALI
KKYPKLESEFVYGDYKVYDVRKMIAKSEQEIGKATAKYFFYSNIMNFFKTEITLANGEIRKRPLIETNGETGEIVWDKGRDFATVRKVLSMPQ
VNIVKKTEVQTGGFSKESILPKRNSDKLIARKKDWDPKKYGGFDSPTVAYSVLVVAKVEKGKSKKLKSVKELLGITIMERSSFEKNPIDFLEAK
GYKEVKKDLIIKLPKYSLFELENGRKRMLASAGELQKGNELALPSKYVNFLYLASHYEKLKGSPEDNEQKQLFVEQHKHYLDEIIEQISEFSKRV
ILADANLDKVLSAYNKHRDKPIREQAENIIHLFTLTNLGAPAAFKYFDTTIDRKRYTSTKEVLDATLIHQSITGLYETRIDLSQLGGDSGGSSGE
FEPKKKRKVGSGATNFSLLKQAGDVEENPGPMVSKGEELIKENMHMKLYMEGTVNNHHFKCTSEGEGKPYEGTQTMRIKVVEGGPLPF
AFDILATSFMYGSRTFINHTQGIPDFFKQSFPEGFTWERVTTYEDGGVLTATQDTSLQDGCLIYNVKIRGVNFPSNGPVMQKKTLGWEAN
TEMLYPADGGLEGRSDMALKLVGGGHLICNFKTTYRSKKPAKNLKMPGVYYVDHRLERIKEADKETYVEQHEVAVARYCDLPSKLGHKLN
*

Supplementary Note 2 | Javascript code for evaluation of the highest A to G conversion within the protospacer

```
const fs = require("fs");

// Configuration

const NON_REVERSE_COMPLEMENT_GUIDE = "CACATATTTTGAAGCAACGG";
const REVERSE_COMPLEMENT = false;
const filename =
  "Nucleotide_percentage_summary_around_sgRNA_CACATATTTTGAAGCAACGG.txt";

// Code (do not need to modify)

const complement = (str) =>
  str
    .split("")
    .map((s) => {
      if (s === "T") {
        return "A";
      } else if (s === "A") {
        return "T";
      } else if (s === "C") {
        return "G";
      } else if (s === "G") {
        return "C";
      }
    })
    .join("");

const createGuide = (str) => (c) => {
  if (c) {
    return complement(str).split("").reverse().join("");
  } else {
    return str;
  }
};

const GUIDE = createGuide(NON_REVERSE_COMPLEMENT_GUIDE)(REVERSE_COMPLEMENT);

const REFERENCE_BASE = createGuide("A")(REVERSE_COMPLEMENT);
```

```
const EDIT_BASE = createGuide("G")(REVERSE_COMPLEMENT);

let data = [];
// use const if the variable never changes and us let if you change it

const create2DArray = (w, h) => {
  let array = new Array(w);
  for (let i = 0; i < w; i++) {
    array[i] = new Array(h);
  }
  return array;
};

fs.readFile(filename, "utf8", (err, string) => {
  const rows = string.split("\n");
  data = create2DArray(rows.length, rows[0].split("\t").length);

  rows.forEach((r, ir) => {
    const columns = r.split("\t");

    columns.forEach((c, cr) => {
      data[ir][cr] = c;
    });
  });

  let startIndex = 2;
  let index = 0;

  let done = false;

  const header = data[0];

  while (!done) {
    if (index > GUIDE.length - 1) {
      done = true;
    } else {
      if (GUIDE[index] === header[startIndex + index]) {
        index += 1;
      } else {
        index = 0;
```

```
      startIndex += 1;

      if (startIndex > data[0].length - 1) {
        console.log("GUIDE Not Found");
        return;
      }
    }
  }
}

data.forEach((r) => {
  if (r[1] === EDIT_BASE) {
    const sliced = r.slice(startIndex, startIndex + GUIDE.length);
    const t = sliced.reduce(
      (acc, v, i) => (GUIDE[i] === REFERENCE_BASE ? acc + Number(v) : acc),
      0
    );

    console.log(`${r[0]}: `, t);
  }
});
});
```

Supplementary Note 3 | Uncropped Western Blot analysis images and PCR Agarose gel images. Top panel: Western blot analysis of data shown in Suppl. Fig. S2. Mouse sgRNAs were tested for the knockdown of PCSK9 in Hepa1-6 cells. (sgRNA1-SD = sgRNA-mP01). Western blot analysis was performed three times for n = 3 biologically independent replicates. Bottom panel: Uncropped gel images of Suppl. Fig. S8.

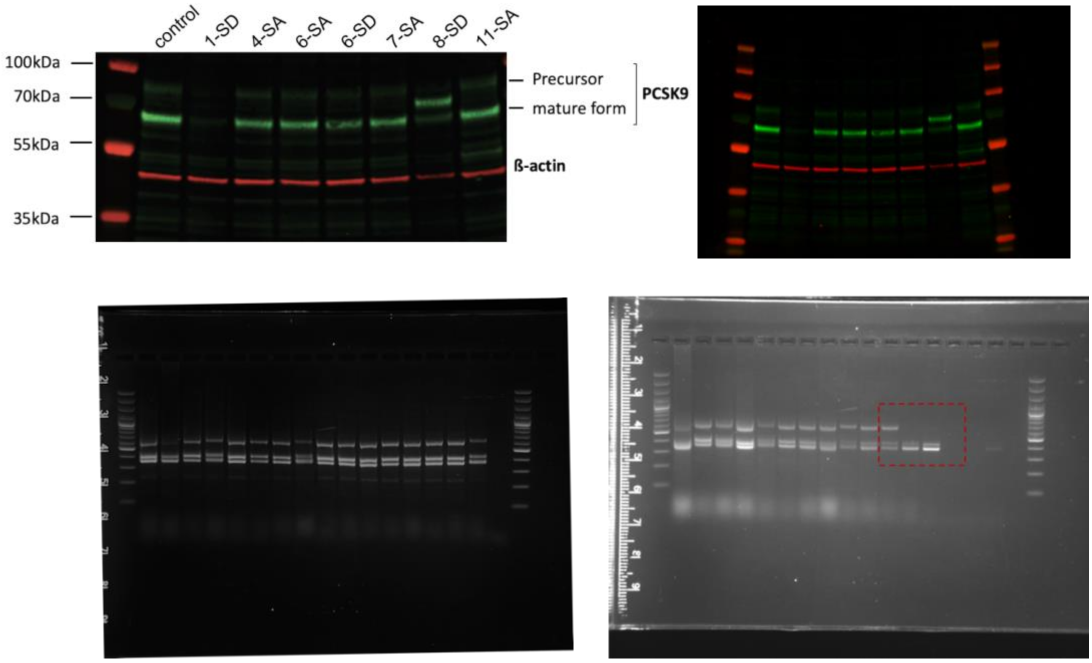

Supplementary References

1. Grünewald, J. *et al.* Transcriptome-wide off-target RNA editing induced by CRISPR-guided DNA base editors. *Nature* (2019). doi:10.1038/s41586-019-1161-z
2. Marquart, K. F. *et al.* Predicting base editing outcomes with an attention-based deep learning algorithm trained on high-throughput target library screens. *bioRxiv* 2020.07.05.186544 (2020). doi:10.1101/2020.07.05.186544
3. Lazzarotto, C. R. *et al.* CHANGE-seq reveals genetic and epigenetic effects on CRISPR–Cas9 genome-wide activity. *Nat. Biotechnol.* (2020). doi:10.1038/s41587-020-0555-7
