# Supplementary material for: In vivo adenine base editing of PCSK9 in macaques reduces LDL cholesterol levels
Source: Nat Biotechnol. 2021 May 19;39(8):949–57. doi: 10.1038/s41587-021-00933-4 (PMC8352781; doi:10.1038/s41587-021-00933-4)
Supplement: Supplementary file 2 — Reporting Summary [file 41587_2021_933_MOESM2_ESM.pdf]

nature research

Corresponding author(s): Gerald Schwank
Last updated by author(s): April 22nd, 2021

Reporting Summary

Nature Research wishes to improve the reproducibility of the work that we publish. This form provides structure for consistency and transparency in reporting. For further information on Nature Research policies, see our Editorial Policies and the Editorial Policy Checklist.

Statistics

For all statistical analyses, confirm that the following items are present in the figure legend, table legend, main text, or Methods section.

n/a	Confirmed	
☐	☒	The exact sample size (n) for each experimental group/condition, given as a discrete number and unit of measurement
☐	☒	A statement on whether measurements were taken from distinct samples or whether the same sample was measured repeatedly
☐	☒	The statistical test(s) used AND whether they are one- or two-sided<br>*Only common tests should be described solely by name; describe more complex techniques in the Methods section.*
☒	☐	A description of all covariates tested
☒	☐	A description of any assumptions or corrections, such as tests of normality and adjustment for multiple comparisons
☐	☒	A full description of the statistical parameters including central tendency (e.g. means) or other basic estimates (e.g. regression coefficient) AND variation (e.g. standard deviation) or associated estimates of uncertainty (e.g. confidence intervals)
☐	☒	For null hypothesis testing, the test statistic (e.g. F, t, r) with confidence intervals, effect sizes, degrees of freedom and P value noted<br>*Give P values as exact values whenever suitable.*
☒	☐	For Bayesian analysis, information on the choice of priors and Markov chain Monte Carlo settings
☒	☐	For hierarchical and complex designs, identification of the appropriate level for tests and full reporting of outcomes
☒	☐	Estimates of effect sizes (e.g. Cohen's d, Pearson's r), indicating how they were calculated

Our web collection on statistics for biologists contains articles on many of the points above.

Software and code

Policy information about availability of computer code

Data collection	Next-generation sequencing (NGS) data was collected and demultiplexed by Illumina NovaSeq Control software (v1.7) and MiSeq Control software (v3.1.); Absorbance measurements were collected with BioTek Synegy HT; RT-qPCR data were collected using QuantStudio™ 5 Real-Time PCR System (Thermo Fisher Scientific) or 7900HT Fast Real-Time PCR System (Applied Biosystems).
Data analysis	NGS data were analyzed by CRISPResso2. Scrips for batch on- and off-target evaluation is added to the Supplement. PE reads were pre-processed using Fastp version 0.20.0 ; PCR-duplicates were marked using Picard version 2.9.0 and Read alignments were comprehensively evaluated using ezRun (https://github.com/uzh/ezRun/). STAR version 2.7.0e was used to map RNA-seq data. Variant calling was performed using GATK v4.1.2.0. using HaplotypeCaller and transcript expression was calculated using kallisto v0.44.0. WGS reads were aligned with BWA v0.7.5. mapping tool; marked for duplicated by using Sambamba v0.4.732 and realigned per donor by using GATK IndelRealigner v2.7.2. Raw variants were multisample-called by using the GATK HeplotypeCAller v3.4-46 and GATK-Queue v3.4-46 and compared to Nucleotide Polymorphism Database v142. The quality of variant and reference positions was evaluated by using GATK VariantFiltration v3.4-46. Diploidy assessment was performed using Control-FREEC v11.6. To exclude in vitro accumulated mutations we used SNVFI and inDelfi software: scripts are available at https://github.com/ToolsVanBox/SNVFI and https://github.com/ToolsVanBox/INDELFI. Called SNVs were compared between groups using the online tool by the van de Peer Lab http://bioinformatics.psb.ugent.be/webtools/Venn/ provided by the VIB/UGent. Cosine similarity with the TadA signature was calculated using MutationalPatterns in Rv4.0.3. The global maximum-likelihood estimates were calculated using dNdScv package v.0.1.0 and plotted using ggplot2 in R v4.0.3. Additional data visualization was performed using Adobe Illustrator v22.1. Images were taken using Zeiss software Zen2 and analyzed by custom ImageJ (v.1.51n). Statistical analyses were performed using GraphPad Prism 6.01 for MacOS. CIRCLE- CHANGE and iGUIDE analysis were done according to the respective publications and statements in the manuscript and were mapped to the following reference genomes: UCSC Macaca_fascicularis_5.0/macFas5; GRCh38/hg38; GRCm39/mm39.

For manuscripts utilizing custom algorithms or software that are central to the research but not yet described in published literature, software must be made available to editors and reviewers. We strongly encourage code deposition in a community repository (e.g. GitHub). See the Nature Research guidelines for submitting code & software for further information.

Data

Policy information about availability of data

All manuscripts must include a data availability statement. This statement should provide the following information, where applicable:

- Accession codes, unique identifiers, or web links for publicly available datasets
- A list of figures that have associated raw data
- A description of any restrictions on data availability

The main data supporting the results is in the study are available within the paper and its Supplementary Information. The raw and analyzed dataasets generated during the study are too large to be publicly shared, yet they are available for research purposes from the corresponding authors on reasonable request. NGS data is publicly available (accession numbers: PRJEB41832 for WGS data, GSE168365 for NGS and RNA-seq data) Additionally used datasets in this study: UniVec (https://www.ncbi.nlm.nih.gov/tools/vecscreen/univec/), refseq mRNA and genome sequences (human, mouse, arabidopsis, bacteria, virus, phix, lambda, mycoplasma: https://www.ncbi.nlm.nih.gov/refseq/), SILVA rRNA sequences (https://www.arb-silva.de/); Single Nucleotide Polymorphism Database (NCBI, v142),

Field-specific reporting

Please select the one below that is the best fit for your research. If you are not sure, read the appropriate sections before making your selection.

☒ Life sciences ☐ Behavioural & social sciences ☐ Ecological, evolutionary & environmental sciences

For a reference copy of the document with all sections, see nature.com/documents/nr-reporting-summary-flat.pdf

Life sciences study design

All studies must disclose on these points even when the disclosure is negative.

Sample size	Sample sizes for in vivo experiments were determined based on literature precedence for genome editing experiments (Villiger 2018, Gaudelli, 2017, Koblan 2021). Further power calculation for treatment in the sensitized background was performed using G*Power (Faul, 2009) aiming for a confidence of 90% to assess a difference in HCC formation.
Data exclusions	No animals and no data were excluded.
Replication	All attempts at replication were successful. Findings were replicated as indicated in the figure legends.
Randomization	Mice were assigned to the treated or untreated groups by litter. No covariates were controlled. For in vitro experiments tno samples were randomized.
Blinding	Researchers were not blinded to group allocation. Blinding was not necessary because no placebo group was present and the readout (editing efficiency, PCSK9 levels, LDL levels) can not be influenced by a biased researcher. All HTS data was analyzed by an unblinded operator by using an automated script (CRISPResso2 + Javascript in Supplement) with limited experimenter intervention.

Reporting for specific materials, systems and methods

We require information from authors about some types of materials, experimental systems and methods used in many studies. Here, indicate whether each material, system or method listed is relevant to your study. If you are not sure if a list item applies to your research, read the appropriate section before selecting a response.

Materials & experimental systems

n/a	Involved in the study
☐	☒ Antibodies
☐	☒ Eukaryotic cell lines
☒	☐ Palaeontology and archaeology
☐	☒ Animals and other organisms
☒	☐ Human research participants
☒	☐ Clinical data
☒	☐ Dual use research of concern

Methods

n/a	Involved in the study
☒	☐ ChIP-seq
☒	☐ Flow cytometry
☒	☐ MRI-based neuroimaging

Antibodies

Antibodies used	mouse-anti-Cas9 mAB (7A9-3A3; clone number:14697T, Cell Signaling, Cat. No#14697); Goat-anti-mouse-HRP (SouthernBiotech, Cat. No. 1030-05) mouse-anti-monkey-HRP (SouthernBiotech, Cat. No. 4700-05); goat anti-Pcsk9 (1:10’000, Cat. No. AF3985-SP; R&D Systems); rabbit anti-beta-actin (1:3’000, Cat. No. #4970S; Cell Signaling) or rabbit anti-GAPDH (1:5’000, Cat. No. 4970; Abcam). HHRP- or IRDye-conjugated secondary antibodies (Donkey anti-Goat: Licor Cat. No. #926-32214; anti-Rabbit: Licor Cat. No. #926-68073 Cell Signaling Cat. No. #7074, Promega Cat. No. #V8051)

Validation	mouse-anti-Cas9 mAB: Validation: https://www.cellsignal.com/products/primary-antibodies/cas9-7a9-3a3-mouse-mab/14697. Validation reference: Borys SM, Younger ST. Identification of functional regulatory elements in the human genome using pooled CRISPR screens. BMC Genomics. 2020 Jan 31;21(1):107. doi: 10.1186/s12864-020-6497-0. PMID: 32005150; PMCID: PMC6995077. Goat-anti-mouse-HRP: Validation: https://www.southernbiotech.com/?catno=1030-05&type=Polyclonal#&panel1-1&panel2-1. Validation reference: van Ginkel FW, Wahl SM, Kearney JF, Kweon M, Fujihashi K, Burrows PD, et al. Partial IgA-deficiency with increased Th2-type cytokines in TGF-β1 knockout mice. J Immunol. 1999;163:1951-7. (ELISA, ELISPOT) mouse-anti-monkey-HRP: Validation: https://www.southernbiotech.com/?catno=4700-05&type=Monoclonal#&panel1-1. Validation reference: Kasturi SP, Skountzou I, Albrecht RA, Koutsonanos D, Hua T, Nakaya HI, et al. Programming the magnitude and persistence of antibody responses with innate immunity. Nature. 2011;470:543-7. (ELISA) goat anti-Pcsk9: Validation: https://www.rndsystems.com/products/mouse-rat-proprotein-convertase-9-pcsk9-antibody_af3985. Validation reference: Kim K, Goldberg IJ, Graham MJ, Sundaram M, Bertaggia E, Lee SX, Qiang L, Haeusler RA, Metzger D, Chambon P, Yao Z, Ginsberg HN, Pajvani UB. γ-Secretase Inhibition Lowers Plasma Triglyceride-Rich Lipoproteins by Stabilizing the LDL Receptor. Cell Metab. 2018 Apr 3;27(4):816-827.e4. doi: 10.1016/j.cmet.2018.02.010. Epub 2018 Mar 22. PMID: 29576536; PMCID: PMC5884729. rabbit anti-GAPDH: Validation: https://www.abcam.com/gapdh-antibody-epr16891-loading-control-ab181602.html. Validation reference: Gao Z et al. Regulatory effects of lncRNA ATB targeting miR-200c on proliferation and apoptosis of colorectal cancer cells. J Cell Biochem 121:332-343 (2020), PMID:31222825. rabbit anti-beta-actin: Validation: https://www.cellsignal.com/products/primary-antibodies/b-actin-13e5-rabbit-mab/4970. Validation reference: Wang N, Yi H, Fang L, Jin J, Ma Q, Shen Y, Li J, Liang S, Xiong J, Li Z, Zeng H, Jiang F, Jin B, Chen L. CD226 Attenuates Treg Proliferation via Akt and Erk Signaling in an EAE Model. Front Immunol. 2020 Aug 21;11:1883. doi: 10.3389/fimmu.2020.01883. PMID: 32983109; PMCID: PMC7478170.

Eukaryotic cell lines

Policy information about cell lines

Cell line source(s)	Hepa1-6 (ATCC CRL-18.30), HEK293T (ATCC CRL-321), HepG2 (ATCC HB-8065)
Authentication	Cell lines were authenticated by the supplier by STR analysis
Mycoplasma contamination	All cells were tested negative for mycoplasma
Commonly misidentified lines (See ICLAC register)	No commonly misidentified cell lines were used

Animals and other organisms

Policy information about studies involving animals; ARRIVE guidelines recommended for reporting animal research

Laboratory animals	Male C57BL/6J mice (study start at 5 weeks of age, 20-25 g of weight)<br>Albumin-Cre x Trp53flox/flox mice, in C57BL/6J background (male and female (M/F) animals: Untreated (17/12); AAV only (16/2), ABE treated (16/9); study start at 5 weeks of age, 20-25 g of weight)<br>Mice were housed in a pathogen-free animal facility at the Institute of Molecular Health Sciences at ETH Zurich, and kept in a temperature- and humidity-controlled room on a 12 hours light-dark cycle.<br>Male Macaca fascicularis (approx. two years of age) were housed in a temperature- and humidity-controlled room on a 12 hours light-dark cycle.
Wild animals	The study did not involve wild animals
Field-collected samples	The study did not involve samples collected from the field
Ethics oversight	Mouse experiments were performed in accordance with protocols approved by the Kantonales Veterinäramt Zürich.<br>Studies involving non-human primates were conducted at a facility accredited by the International Laboratory Animal Assessment and Accreditation (AAALAC), operating in accordance with the principles of US FDA's Good Laboratory Practice (GLP) and the Guide for the Care and Use of Laboratory Animals, Institute of Laboratory Animal Resources, National Academy Press, Washington, D.C., 2011. All protocols were reviewed and approved by the Acuitas animal care and use committee.

Note that full information on the approval of the study protocol must also be provided in the manuscript.
